# Simulating Thermoresponsive Behavior of Disordered Proteins with Temperature-Dependent Coarse-Grained Potentials Derived from Hydration Free Energies

**DOI:** 10.1021/jacsau.6c00523

**Published:** 2026-08-03

**Authors:** Fangke Chen, Xiangze Zeng

**Affiliations:** † Department of Physics, 26679 Hong Kong Baptist University, Kowloon Tong, Hong Kong 999077, China; ‡ The Computational Medicine Lab, School of Chinese Medicine, Hong Kong Baptist University, Kowloon Tong, Hong Kong 999077, China

**Keywords:** phase separation, lower critical solution temperature, disordered proteins, coarse-grained simulations, hydration free energies, temperature-dependent interactions

## Abstract

Thermoresponsive phase transitions of intrinsically disordered proteins (IDPs), including both upper critical solution temperature (UCST) and lower critical solution temperature (LCST) transitions, are widely observed in natural and synthetic sequences. However, most existing coarse-grained (CG) models employ temperature-independent interactions and fail to capture solvation-driven LCST behavior. Here, we bridge the gap between atomistic hydration and macroscopic phase separation. Leveraging 500 μs long extensive all-atom molecular dynamics simulations, we reveal a fundamental linear correlation between the temperature-dependent inter-residue interaction strengths and hydration free energies. Furthermore, we demonstrate that the heterotypic interactions at the molecular level can be well approximated by a simple combination of the homotypic interactions. We incorporate these thermodynamic insights into a physics-based framework, **TEA** (**T**emperature-dependent **E**nergetics derived from hydr**A**tion), which introduces temperature-dependent potentials with minimal phenomenological fitting. The TEA-augmented CG models robustly distinguish UCST- and LCST-type sequences, successfully identify experimentally reported outliers, and accurately reproduce LCST-type single-chain compaction trends and phase diagrams of multiple disordered proteins. Our work provides a transferable and physically interpretable framework that bridges atomistic hydration thermodynamics and phase behavior of IDPs, enabling the simulation of thermoresponsive sequences with minimal phenomenological fitting.

## Graphical abstract

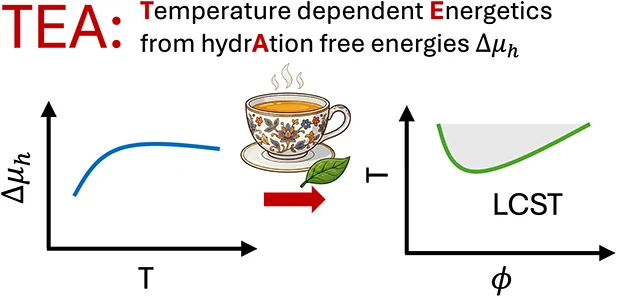

## Introduction

Biomolecular phase separation has emerged as a fundamental principle for understanding various cellular processes.^1–4^ Recent studies have demonstrated the crucial roles of intrinsically disordered proteins or regions (IDPs) as drivers or regulators of cellular phase separation, owing to their multivalent interactions.^5–9^ Unlike folded proteins, IDPs lack stable tertiary structures and instead sample a heterogeneous ensemble of conformations dictated by their low fractions of hydrophobic residues. This structural flexibility enables IDPs to function as flexible linkers between folded domains, molecular recognition motifs for protein binding, and molecular assemblers promoting higher-order structure formation.^10^ Consequently, elucidating the conformational ensembles and phase behaviors of IDPs is crucial for understanding the molecular basis of their biological functions.

Molecular simulations, including molecular dynamics (MD) and Monte Carlo methods, are essential tools for probing protein dynamics at high spatiotemporal resolution.^11–13^ However, atomistic simulations of IDPs face two major challenges. First, widely used protein force fields are typically parametrized for folded proteins, leading to overly collapsed states when applied to IDPs.^14–16^ To mitigate this issue, various modifications have been made to these force fields.^17–23^ For example, Best et al. scaled the protein–water interactions in the Amber ff03 force field to prevent overcompaction.^17^ Besides modifying protein–water interactions, Robustelli et al. refined torsion parameters in Amber 99sb-ILDN force field to balance helix and coil populations.^18^ Huang et al. introduced an improved CMAP backbone potential in CHARMM36m force field.^19^ The second challenge is the high computational cost for simulating extended IDP conformations, requiring large simulation boxes with explicit water molecules that significantly slow down simulation speed. An approach to address this challenge is using an implicit solvent model. For instance, the ABSINTH all-atom model has been developed and parametrized for IDPs.^24^ While these all-atom force fields have achieved some success in capturing the single-chain conformations of IDPs, simulating the phase separation involving at least hundreds of IDPs remains computationally challenging at the atomistic level.

To bridge these spatiotemporal scales, coarse-grained (CG) simulations have been widely adopted for studying IDP phase separation.^25–35^ For instance, Choi et al. developed the phenomenological lattice model LaSSI, which can capture the essential underlying physical principles of protein phase separation.^25,36^ Dignon et al. developed the one-bead-per-residue CG model HPS based on the residue hydrophobicity scale.^26^ Tesei et al. further improved the parameters of the HPS model using a data-driven approach and proposed the CALVADOS model.^27^ Similarly, Joseph et al. developed the mpipi model using both atomistic simulations and bioinformatic data.^28^ These models successfully capture phase separation with an upper critical solution temperature (UCST), where phase separation is driven by favorable enthalpy upon cooling.

However, these models fail to describe phase separation with a lower critical solution temperature (LCST), where phase separation occurs upon heating. This limitation arises from the temperature-independent interresidue energies in these implicit solvent models. One physical origin of LCST-type phase behavior is the solvation effect, where residues become less favorably solvated at higher temperatures.^37,38^ Meanwhile, Wadsworth et al. showed that ion effects are another driving force for LCST-type phase separation.^39^ Specifically, they found that more Mg^2+^ ions bridge RNA molecules by binding to multiple phosphate groups at elevated temperatures, which drives phase separation. In both cases, the effective interactions between solute molecules increase with temperature. Since these CG models employ implicit solvents, they cannot fully capture these solvation effects or ion effects.

The all-atom implicit solvent model ABSINTH has been shown to reproduce the LCST-type phase behavior by including a temperature-dependent solvation term in the Hamiltonian.^37^ Wuttke et al. incorporated experimentally inferred temperature-dependent solvation free energies into ABSINTH and observed the LCST-type single-chain coil-to-globule transitions for five IDPs.^37^ Building on this, we previously calculated temperature-dependent hydration free energies for the backbone and side chain analogues from all-atom simulations with the polarizable force field AMOEBA^40^ and developed the ABSINTH-T model.^38^ Combined with a genetic algorithm, we successfully designed synthetic IDPs with LCST-type phase behavior, which have been validated by preliminary experimental measurements.^38^

Several attempts have been made to incorporate temperature-dependent potentials into coarse-grained models.^41–43^ Based on the HPS model, Dignon et al. used a parabolic function to describe the temperature-dependent interaction parameters λ.^41^ The final parameters were obtained by fitting the single-chain coil-to-globule transitions of five LCST-type IDPs from Wuttke et al.’s experimental study.^37^ Recently, Chakravarti and Joseph used a data-driven approach to fit the coarse-grained temperature-dependent interactions.^42^ Specifically, building on the insights of Wuttke et al.^37^ and the ABSINTH-T model,^38^ they used the functional form of hydration free energies to fit the temperature-dependent interaction strengths in the mpipi model. These efforts support that integrating the temperature-dependent potentials into the coarse-grained models can capture the LCST-type phase behavior. However, while experimental data for LCST-type phase behavior is limited, it is still challenging to obtain consistent temperature-dependent energetics across residue chemistries, construct heterotypic interactions without an explosion of fitted parameters, and map atomistic thermodynamics onto CG potentials in a way that is both predictive and minimally overfit.

In this study, we introduce TEA (Temperature-dependent Energetics derived from hydrAtion free energies), a physics-based framework that augments coarse-grained models with temperature-dependent potentials based on hydration free energies. Our approach includes three stages. First, we obtain the potential of mean forces between amino acid side chain/backbone analogues from extensive all-atom MD simulations and quantify the temperature-dependent interactions. The accumulated simulation time for all-atom MD simulations is over 500 μs. Using these simulation data, we demonstrate that the interaction strengths of the interacting species exhibit a linear dependence on their hydration free energies. This linearity allows us to get the interaction strengths in the continuous temperature space. Moreover, we show that the heterotypic interactions among different species can be well approximated from the homotypic interactions using a combination rule, which enables scalable construction of a full interaction matrix. In the second stage, we leverage these physical insights and map the atomistic energetics to CG potentials via a linear relationship, which is governed by a single global scaling parameter, γ. This single free parameter allows us to integrate TEA into existing coarse-grained models with minimal reparameterization. Lastly, we fitted γ and validated this framework against independent experimental data sets. We demonstrate that the TEA-augmented CG potentials provide a transferable, physically interpretable route to simulate thermoresponsive IDPs with significant predictive power, successfully capturing LCST phase behavior and identifying sequence-specific outliers without relying on extensive parameter fitting to experimental data.

## Results

### Temperature-Dependent Homotypic Interactions for Amino Acid Side Chain Analogues Were Obtained from All-Atom MD Simulations

To establish a thermodynamic basis for coarse-grained interactions, we quantified the temperature-dependent interactions between amino acid side chain/backbone analogues using extensive MD simulations. The model compounds used to mimic amino acid side chain and backbone are listed in Table S1. The potential of mean forces (PMFs) for interacting pairs were obtained from umbrella sampling. We utilized the CHARMM36 force field^19,44–46^ to get PMFs for homotypic pairs across a temperature range of 280 to 380 K (Figure S1). More details of the simulations can be found in the Methods.

For hydrophobic analogues, such as Leu and Ile, the interaction strength increases with temperature, evidenced by a deepening minimum in the PMF curves (Figure S1). Conversely, hydrophilic analogues, such as Ser and Thr, exhibit negligible temperature dependence (Figure S1). For charged analogues, we isolated the solvent-mediated van der Waals (vdW) contributions by subtracting the direct Coulombic interaction ( $U_{\mathrm{Coulomb}}=\frac{q_{i}q_{j}}{4\pi \varepsilon_{0}\varepsilon_{r}r_{ij}}$ with temperature-dependent ε_r_) from the calculated PMF. The resulting PMFs after subtraction quantify the vdW interactions mediated by water molecules between the charged analogues. It is important to note that while we subtract the analytical Coulomb term, the direct electrostatic interactions still implicitly influence the resulting PMFs by affecting the water arrangement between the charged molecules. The goal of this decoupling is specifically to derive the coarse-grained force field parameters of the modified Lennard-Jones (LJ) potential of charged species, independent of the long-range electrostatics handled separately in the CG model. The resulting PMFs confirm the hydrophilic nature of charged analogues, showing minimal variation with temperature (Figure S1).

To translate these PMFs into a metric suitable for CG parametrization, we defined the excess free energy (Δ*G*_E_) for an interacting pair:

1 $$\Delta G_{E}=-k_{B}T\times \ln\frac{\int_{0}^{r_{c}}\exp\left(-\frac{W\left(r\right)}{k_{B}T}\right)\times 4\pi r^{2}dr}{\int_{0}^{r_{c}}4\pi r^{2}dr}$$

where *W*(*r*) is the corresponding PMF, *r* is the intermolecular distance, *k*_B_ is the Boltzmann constant, and the cutoff *r*_c_ is set to 1.0 nm. Δ*G*_E_ provides a direct measure of the intrinsic interaction strength relative to a noninteracting ideal gas within the same volume, allowing for direct comparison across different chemical species. We note that the definition for Δ*G*_E_ is formally analogous to the standard binding free energy defined as:

$$\Delta G_{\mathrm{bind}}^{0}=-k_{B}T\times \ln\left(\frac{1}{V^{0}}\int_{0}^{r_{c}}\exp\left(-\frac{W\left(r\right)}{k_{B}T}\right)\times 4\pi r^{2}dr\right),$$

provided the region *r* < *r*_c_ is defined as the bound region. *V* ^0^ is the standard state volume 1.661 nm^3^, corresponding to a 1 M concentration. Consequently, Δ*G*_E_ and $\Delta G_{\mathrm{bind}}^{0}$ differ only by a molecule-independent value. Δ*G*_E_ is therefore useful for calibrating the relative interaction energies required for fitting CG potential parameters.

Figure 1 shows Δ*G*_E_ as a function of temperatures for different homotypic interactions. As expected, Δ*G*_E_ decreases substantially for hydrophobic analogues (e.g., Ile and Phe) as temperature rises, indicating stronger attractions, whereas it shows no significant changes for hydrophilic residues (e.g., Ser and Gln). We further compared values of Δ*G*_E_ at 300 K for side chain analogues with the corresponding hydrophobicity scales in several prominent CG models for simulating IDP phase separation. We found that Δ*G*_E_ (300 K) show high correlations with existing scales (Figure S2). The correlation coefficients with HPS, HPS-Urry, HPS-FB, CALVADOS (M2) (the M2 parameters), mpipi, and mpipi-Recharged^47^ are 0.63, 0.85, 0.65, 0.79, 0.64, and 0.62, respectively (Figure S2). We note that the HPS model assigns a λ value of zero to Arg, the key parameter indicating the van der Waals interaction strength with other residues. This assignment makes Arg a clear outlier in the Δ*G*_E_ (300 K) v.s. λ plot shown in Figure S2. Without considering the charged species, Δ*G*_E_ (300 K) shows stronger correlations with these models, with coefficients of 0.78, 0.85, 0.70, 0.73, 0.69, and 0.66 for HPS, HPS-Urry, HPS-FB, CALVADOS (M2), mpipi, and mpipi-Recharged, respectively. Interestingly, correlations with the more physics-driven models like HPS-Urry and HPS are higher than those with more data-driven models such as HPS-FB.

**Figure 1:**
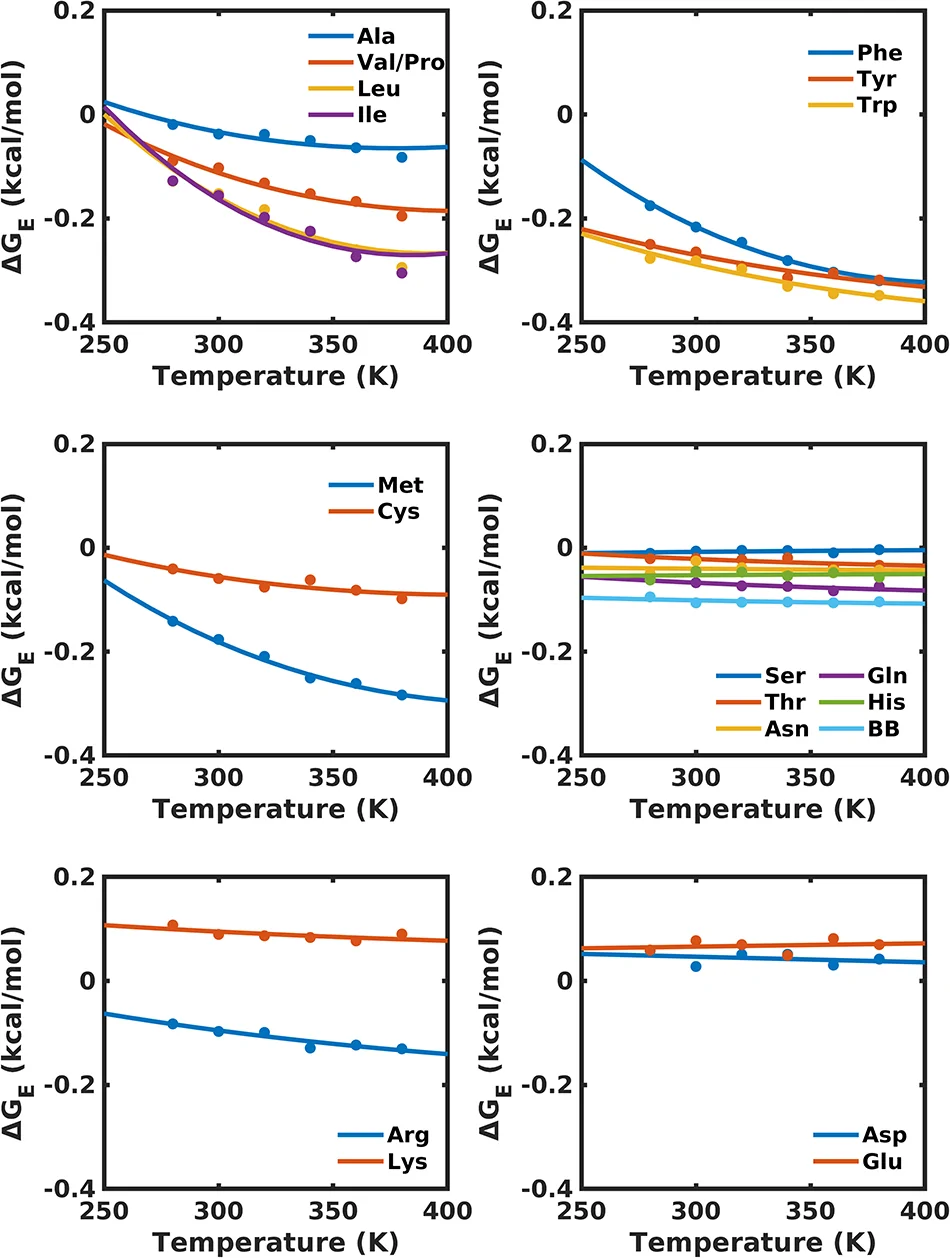
Temperature-dependent excess free energies Δ*G*_E_ for amino acid side chain and backbone analogues. Solid spheres represent results calculated from potential of mean forces (PMFs), while solid lines indicate fits obtained by combining the integrated Gibbs–Helmholtz equation (eq 2) and with the linear scaling relationship (eq 3).

### Excess Free Energy ΔG_E_ Can Be Derived from Hydration Free Energy Δu_h_

While umbrella sampling provides interaction data at discrete temperatures, a robust CG model requires continuous temperature dependence. All-atom models with implicit solvents, such as EEF1 ^48^ and ABSINTH,^24^ account for temperature-dependent interactions by introducing a solvation term into the Hamiltonian. Inspired by this approach, we asked if Δ*G*_E_ can be approximated using hydration free energy.

Next, we calculated the hydration free energies Δ*u*_h_ for all analogous molecules at temperatures from 280 to 380 K (Figure 2). We fitted these data to the integrated version of the Gibbs–Helmholtz equation and obtained the temperature-dependent Δ*u*_h_:

2 $$\Delta u_{h}\left(T\right)=\frac{\left[\Delta u_{h}\left(T_{0}\right)-\Delta h\right]T}{T_{0}}+\Delta h+\Delta c_{p}\left[T\left(1-\ln\frac{T}{T_{0}}\right)-T_{0}\right]$$

**Figure 2:**
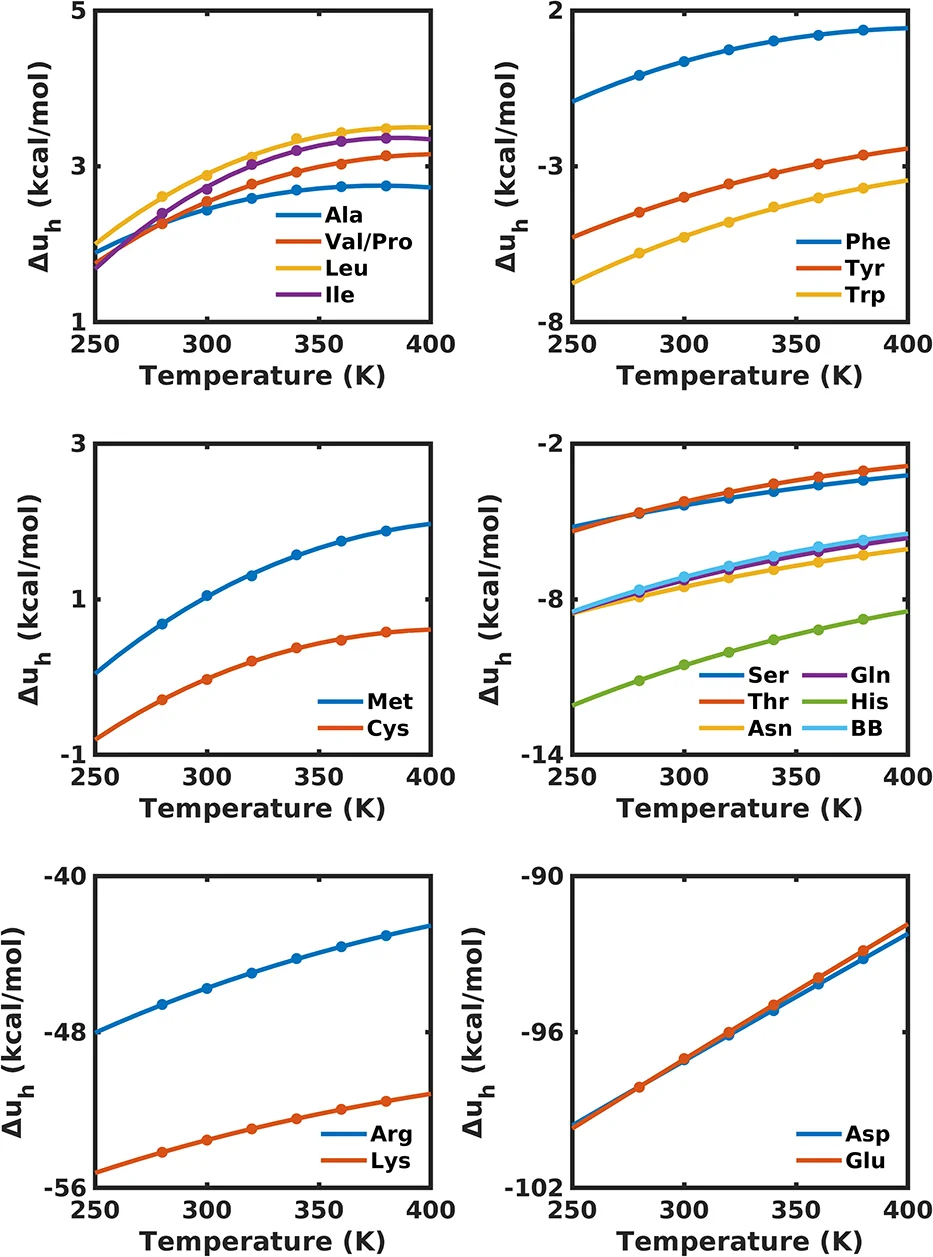
Temperature-dependent hydration free energies Δμ_h_ for amino acid side chain and backbone analogues. Solid spheres represent results from free energy calculations in explicit solvent, and solid lines are the fit to the integrated Gibbs–Helmholtz equation (eq 2).

Here, Δ*h* is the hydration enthalpy at a reference temperature *T*_0_, Δ*c*_p_ is the change of molar heat capacity associated with the hydration process. *T*_0_ was set to 300 K. The fitted parameters for Δ*u*_h_(*T*_0_), Δ*c*_p_, and Δ*h* are shown in Table S1. The fitted temperature-dependent Δ*u*_h_(*T*) are shown by solid curves in Figure 2.

We compared our calculated hydration thermodynamic parameters (μ_h_(*T*_0_), Δ*h*, Δ*c*_p_) with experimental values from Makhatadze and Privalov^49^ and the ABSINTH parameters^24^ used by Wuttke et al.^37^ (Figure S4). For neutral analogues, we observe excellent agreement. For charged analogues, while absolute values deviate due to the inherent experimental uncertainties in parsing single-ion solvation energies, our model accurately captures the fundamental physical trends:^50^ anions are more favorably solvated than cations, and Lys is significantly more favorably solvated than Arg. These trends align perfectly with recent polarizable force field calculations and results from a thermodynamic cycle analysis.^50^ In particular, when the proton hydration free energy is selected as −246 kcal/mol in the thermodynamic cycle approach in Fossat et al.,^50^ the resultant Δvalues for charged species show an exceptionally strong correlation with values calculated in this study (Figure S4).

A comparison of Δ*G*_E_ and Δ*u*_h_ reveals a striking linear correlation across all analogous molecules (Figure 3A). This relationship allows us to express the interaction strength as a function of hydration free energies:

3 $$\Delta G_{E}^{\left(i\right)}\left(T\right)-\Delta G_{E}^{\left(i\right)}\left(T_{0}\right)=\kappa_{i}\left[\Delta u_{h}^{\left(i\right)}\left(T\right)-\Delta u_{h}^{\left(i\right)}\left(T_{0}\right)\right]$$

where κ_*i*_ is the residue-specific coefficient, quantifying the sensitivity of homotypic interactions to hydration changes. The mean absolute error of using eq 3 to fit the data is 0.007 kcal/mol. Values of κ_*i*_ and correlation coefficients *r*_*i*_ are listed in Table 1. Notably, for analogues with |κ_*i*_| > 0.01, the corresponding |*r*_*i*_| values range from 0.88 to 0.98 (Figure 3C), demonstrating a strong linearity between Δ*G*_E_ and Δ*u*_h_. However, it is important to note that the correlation coefficient is not always a reliable measure of linearity when the slope (κ_*i*_) is near zero. Specifically, for analogues with |κ*_i_* |<0.01, *r*_*i*_ spans from −0.86 to 0.50, while the mean absolute errors for these data points remain within 0.005 kcal/mol.

**Figure 3:**
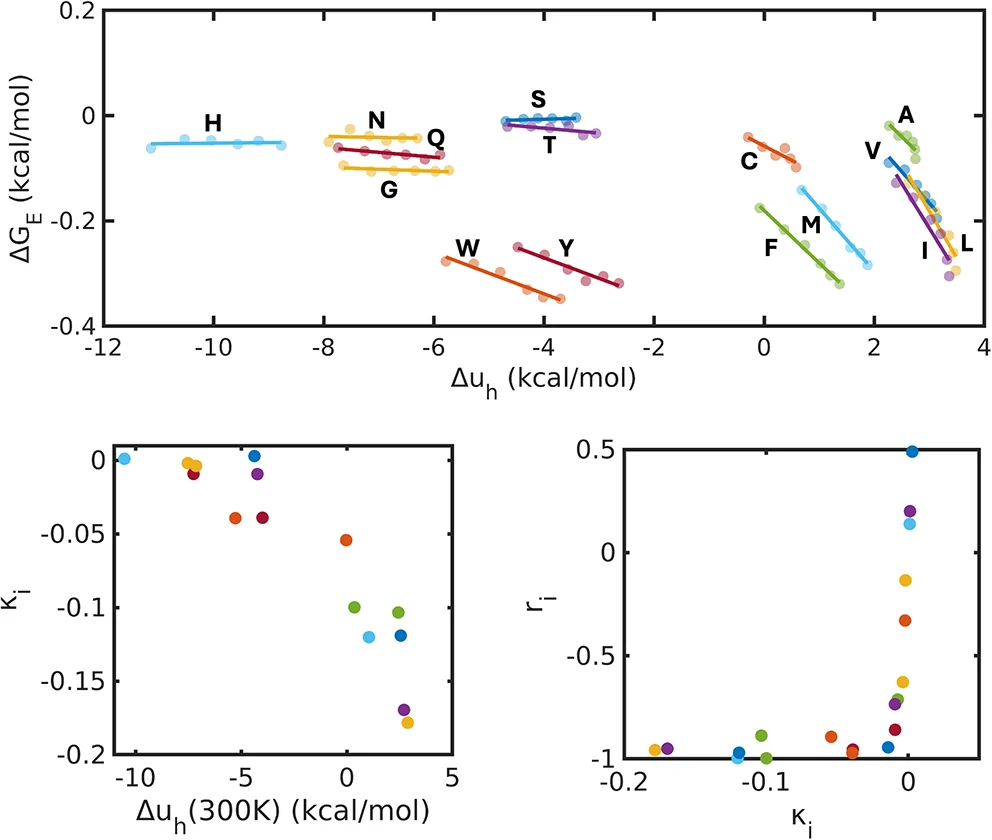
Excess free energies Δ*G*_E_ exhibit a linear relationship with hydration free energies Δ*u*_h_. (A) Δ*G*_E_ plotted against Δ*u*_h_ for neutral amino acid side chain and backbone analogues. Solid spheres represent all-atom simulation data, and solid lines are linear fits using eq 3. Data for charged species are provided in Figure S3. (B) The residue-specific coefficients κ_*i*_ plotted as a function of hydration free energies at 300 K Δ*u*_h_ (300 K). (C) Dependence of the correlation coefficients *r*_*i*_ on the slope κ_*i*_.

**Table 1:** Linear Regression Parameters Correlating Excess Free Energies (Δ*G*_E_) with Hydration Free Energies (Δ*u*_h_)

Residue/unit	κ_*i*_	*r*_*i*_	Mean absolute error (kcal/mol)
Ala	–0.103	–0.887	0.008
Val/Pro	–0.119	–0.970	0.008
Leu	–0.178	–0.957	0.015
Ile	–0.170	–0.951	0.017
Met	–0.120	–0.996	0.004
Phe	–0.100	–0.997	0.003
Cys	–0.054	–0.893	0.006
Tyr	–0.039	–0.955	0.006
Trp	–0.039	–0.971	0.006
Ser	0.003	0.489	0.002
Thr	–0.009	–0.735	0.004
Asn	–0.002	–0.134	0.006
Gln	–0.009	–0.859	0.003
His	0.001	0.139	0.006
Backbone/Gly	–0.004	–0.628	0.002
Arg	–0.014	–0.944	0.005
Arg (charmm36)	0.059	0.976	0.012
Lys	–0.007	–0.712	0.006
Asp	–0.002	–0.329	0.010
Glu	0.001	0.201	0.009

Figure 3A shows that hydrophobic analogues with relatively high Δ*u*_h_ values exhibit strongly negative κ_*i*_ values, confirming that their temperature-dependent attraction is driven by the entropic gain of dehydration. In contrast, hydrophilic analogues show κ_*i*_ values near 0, suggesting a weak dependence of interaction strength on hydration. Although there is a strong linear relationship between Δ*G*_E_ and Δ*u*_h_ for individual species, the relationship across different species is complex. Specifically, we cannot use a single universal κ value to connect Δ*G*_E_ and Δ*u*_h_ for all analogues.

Figure 3B shows the relationship between κ_*i*_ and Δ*u*_h_ (300 K) for noncharged analogues. Here, Δ*u*_h_ (300 K) reflects the hydrophobicity for each analogue. As the analogue becomes more hydrophilic, the dependence of Δ*G*_E_ on Δ*u*_h_ weakens, with κ_*i*_ approaching 0 when Δ*u*_h_ (300 K) falls below −6 kcal/mol. This suggests that in the highly hydrophilic regime, interactions become effectively temperature independent. Since eq 3 is validated by results from all-atom molecular dynamics simulations with explicit solvents, solvation effects are considered in the interactions regardless of the κ_*i*_ value. Additionally, in this regime, the change in Δ*u*_h_ from 280 to 380 K is comparable to or even larger than that in the hydrophobic regime, demonstrating a decoupling between Δ*G*_E_ and Δ*u*_h_. Furthermore, the data points in Figure 3B appear to follow a master curve, suggesting the existence of a general physical principle governing the relationship between κ_*i*_ and Δ*u*_h_ (300 K).

By combining eqs 2 and 3, $\Delta G_{E}^{\left(i\right)}\left(T\right)$ can be quantified within the continuous temperature space. Using the parameters for Δ*u*_h_ from all-atom simulations (Table S1) and the residue-specific κ values (Table 1), the strength of homotypic interactions can be directly obtained from hydration free energies.

### Heterotypic Interactions Are Accurately Predicted by a Simple Combination Rule

Although homotypic interactions can be calculated on the basis of hydration free energy, constructing the full interaction matrix for all residue pairs via umbrella sampling is computationally intractable. In all-atom MD simulations, combination rules are commonly used to approximate nonbonded heterotypic interactions between different atoms.^51^ However, it remains unclear whether these simple rules are valid at the molecular level. Many CG models, such as Martini 2,^52^ HPS-FB,^31^ and CALVADOS,^27^ assume the validity of combination rules in their parametrization. Conversely, models like Martini 3 ^53^ and mpipi^28^ employ pair-specific parameters for heterotypic interactions, allowing more parametrization flexibility. To address this uncertainty directly, we performed large-scale molecular simulations to evaluate whether heterotypic interactions among analogues can be approximated from homotypic data by using standard combination rules.

We computed PMFs for 43 randomly selected heterotypic pairs at 300 K (Figures S5–S9). These 43 pairs cover 5 distinct interaction types: hydrophilic–hydrophilic, hydrophobic–hydrophilic, hydrophobic–hydrophobic, charged–charged, and cation-aromatic interactions (Table S3). We then compared the directly calculated excess free energy, $\Delta G_{E}^{\left(i,j\right)}$ , for a pair *i* and *j* against the arithmetic mean of their homotypic counterparts, $(\Delta G_{E}^{\left(i\right)}+\Delta G_{E}^{\left(j\right)})/2$ .

To calculate Δ*G*_E_ from corresponding PMF curves, a uniform cutoff distance of 10 Å was used for all analogues. We note that within this range, the PMF curves generally exhibit a metastable state for most pairs indicated by a local minimum (Figure S1), corresponding to the solvent-mediated state without direct contact. This state is similar to the solvent-share ion pair (SIP) state observed in cation–anion interactions.^54^ Applying a uniform cutoff yields a linear relationship between Δ*G*_E_ and the binding affinity $\Delta G_{b}^{0}$ with a constant offset, confirming the robustness of this definition.

We subsequently evaluated a molecule-specific cutoff defined by the first energy barrier, which corresponds to the region of direct contact of the molecule pair. As shown in Figure S10, the resulting excess free energies $\left(\Delta G_{E}^{\prime }\right)$ at 300 K correlate strongly with those derived using the 10 Å cutoff. The $\Delta G_{E}^{\prime }$ versus Δ*u*_h_ plot is shown in Figure S11, with fitted parameters listed in Table S2. Notably, the residue-specific scaling factor $\kappa_{i}^{\prime }$ connecting $\Delta G_{E}^{\prime }$ and Δ*u*_h_ is similar to those using a uniform cutoff (Figure S11D). Furthermore, the $\kappa_{i}^{\prime }$ versus Δ*u*_h_ (300 K) plot in Figure S11B follows a similar master curve shown in Figure 3B and the combination rule holds as well as shown in Figure 4B.

**Figure 4:**
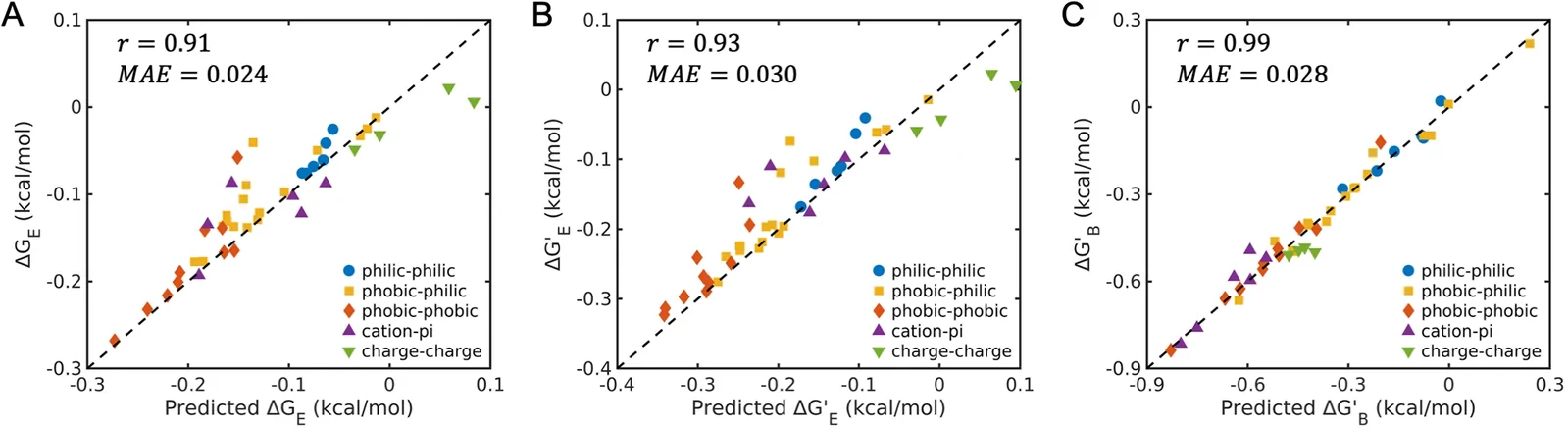
Validation of the combination rule for predicting heterotypic interactions. The plot compares values predicted using the combination rule (*x*-axis) against values calculated directly from all-atom simulations (*y*-axis) for (A) excess free energies Δ*G*_E_ calculated using a uniform cutoff of 10 Å, (B) excess free energies $\Delta G_{E}^{\prime }$ calculated using molecule-specific cutoffs, and (C) binding free energies $\Delta G_{B}^{\prime }$ calculated using molecule-specific cutoffs. Molecule-specific cutoff values are listed in Table S2. Data points are colored by interaction type: hydrophilic–hydrophilic (blue), hydrophobic–hydrophilic (red), hydrophobic–hydrophobic (yellow), cation-π (purple), and charge–charge (green). The dashed line represents perfect agreement (*y* = *x*).

Surprisingly, when the residue-specific cutoff distance is used to calculate the binding free energy

$$\Delta G_{B}^{’}=-k_{B}T\times \ln\left(\frac{1}{V^{0}}\int_{0}^{r_{c,i}}\exp\left(-\frac{W\left(r\right)}{k_{B}T}\right)\times 4\pi r^{2}dr\right),$$

where *r*_c,*i*_ is the distance cutoff only considering the direct contact, the combination rule exhibits exceptionally high fidelity (Figure 4C). While the physical origin of this improved correlation warrants further investigation, the robustness of the results suggests our energetic derivation is not an artifact of the cutoff choice.

As shown in Figure 4, the direct calculations and the arithmetic means are highly correlated. This confirms that heterotypic interactions can be accurately approximated by using a simple combination rule. Consequently, the full matrix of temperature-dependent interactions can be constructed solely from the hydration free energies of individual analogues, bypassing the need for exhaustive pairwise sampling.

### Mapping Temperature-Dependent Atomistic Energetics to Coarse-Grained (CG) Potentials

We next aimed to integrate the temperature-dependent atomistic energetics into CG potentials. We chose the Hamiltonian form common to HPS^26^ and HPS-based models.^27,31,55^

4 $$E_{\mathrm{total}}=E_{\mathrm{bonded}}+E_{vdw}+E_{ele}$$

*E*_vdw_ is represented by the Ashbaugh-Hatch function:

5 $$E_{vdw}=\{\begin{array}{l} E_{LJ}+\left(1-\lambda \right)\epsilon ,\;\mathrm{if}\;r<2^{1/6}\sigma \\ \lambda E_{LJ},\;\mathrm{otherwise} \end{array}$$

*E*_LJ_ is the standard Lennard-Jones potential. ϵ is 0.2 kcal/mol. σ is the van der Waals radius. The critical parameter in these models is the residue-specific stickiness parameter λ. A larger λ value represents stronger attractions. In the HPS and HPS-based models, λ values are constant across different temperatures. Temperature-dependent λ values were introduced using a parabolic function form in the HPS-T model.^41^

To introduce temperature dependence, we mapped the atomistic Δ*G*_E_ to the CG parameter λ via a linear transformation governed by a single global scaling factor γ:

6 $$\lambda \left(T\right)-\lambda \left(T_{0}\right)=\gamma \left[\Delta G_{E}\left(T\right)-\Delta G_{E}\left(T_{0}\right)\right]$$

Here, *T*_0_ is the reference temperature, 300 K. γ is a universal coefficient for all analogues with a unit of (mol/kcal). It accounts for the difference in energetics between the all-atom model and the coarse-grained model. This formulation allows us to augment existing HPS-based models, such as HPS-Urry,^55^ HPS-FB,^31^ and CALVADOS,^27^ with a physics-based temperature dependence while retaining their original parametrization λ at the reference temperature *T*_0_. Our approach is similar to the recently developed mpipi-T model,^42^ which fitted linear coefficients between λ and Δ*u*_h_ using experimental data. Additionally, γ values for heterotypic interactions in mpipi-T are fitted as well instead of being obtained using the combination rule. However, our method leveraged physical insights derived directly from extensive all-atom MD simulations to get the residue-specific κ_*i*_, leaving only a single free parameter γ to be fitted against experiment. This minimizes the risk of overfitting and ensures transferability. We term our approach TEA, abbreviated for “Temperature-dependent Energetics derived from hydrAtion free energies”.

### TEA-Augmented CG Models Robustly Capture Thermoresponsive Phase Behavior of IDPs

We next incorporated TEA into three CG models: HPS-Urry,^55^ HPS-FB^31^ and CALVADOS (M2).^27^ Then, we calibrated the scaling factor γ using experimental data on the thermoresponsive behaviors of resilin-like polypeptides (RLPs) and elastin-like polypeptides (ELPs) reported by Quiroz and Chilkoti.^56^ RLPs and ELPs exhibit UCST- and LCST-type phase separation behaviors, respectively. Previous studies have demonstrated a strong coupling between single-chain conformations and the phase separation behaviors of IDPs.^57–63^ Specifically, IDPs with UCST-type behavior expand at higher temperatures, whereas LCST-type IDPs collapse as temperature increases. Based on this relationship, we performed single-chain simulations for 23 RLP sequences and 16 ELP sequences across temperatures from 250 to 400 K.

We found that all three TEA-augmented CG models, including CALVADOS (M2), HPS-Urry, and HPS-FB, achieved a clear separation between RLP and ELP sequences when γ was set between 2 and 3 (Figures 5 and S12 and S13). As shown in Figure 5, the radius of gyration (*R*_g_) for RLP sequences increases with temperature, while ELP sequences undergo compaction, consistent with their respective UCST- and LCST-type phase behavior. To have a quantitative assessment of the fitting quality, we compared the simulated single-chain *R*_g_ values at 350 K of these sequences with their cloud point temperatures (*T*_cloud_) observed in experiments. As expected, we observed a positive correlation between the single-chain *R*_g_ values and *T*_cloud_ for the ELP sequences and a negative correlation for the RLP sequences (Figures 5C–D and S12–S14).

**Figure 5:**
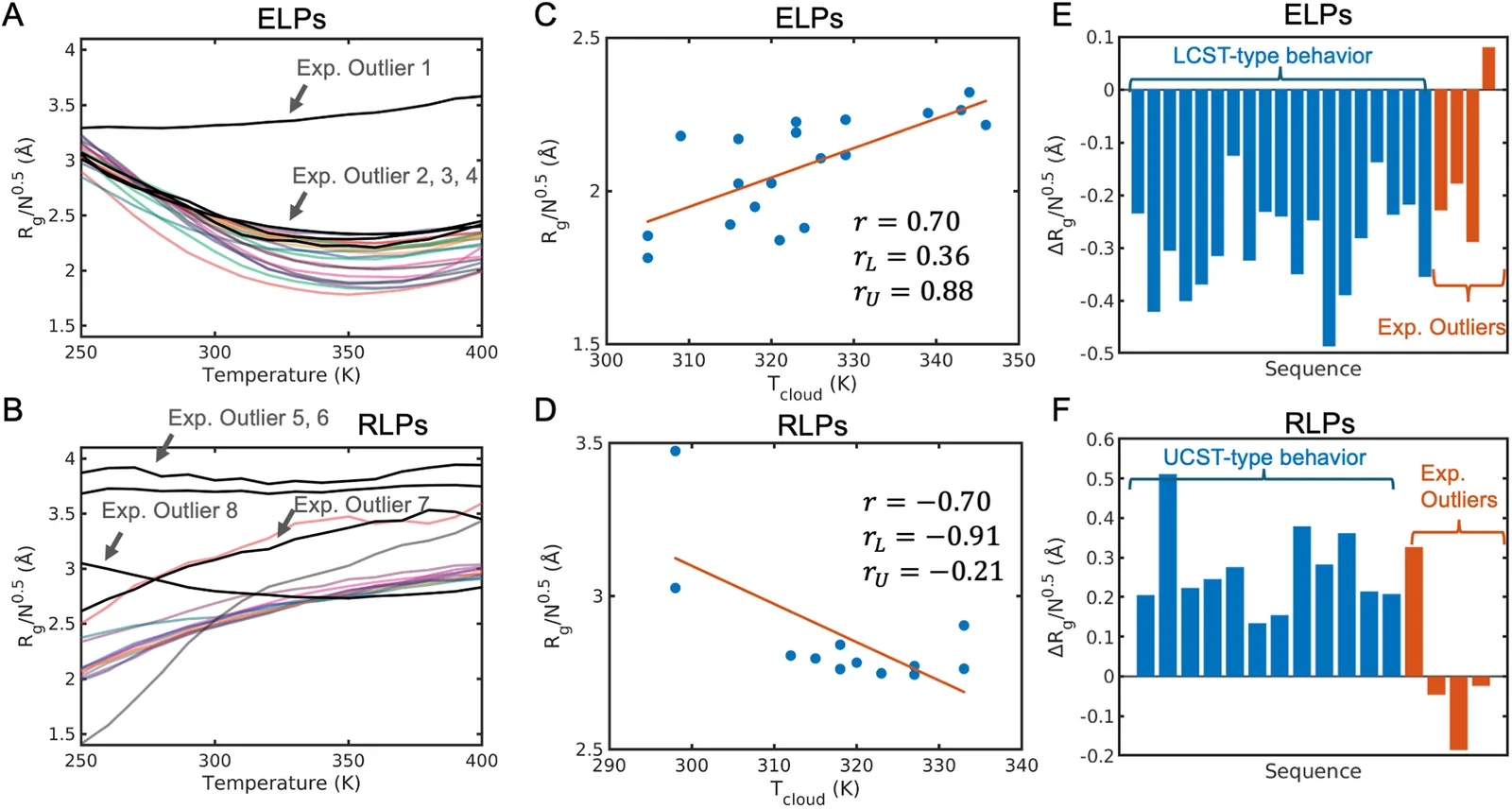
TEA-augmented HPS-Urry model distinguishes between LCST-type phase behavior in elastin-like polypeptides (ELPs) and UCST-type phase behavior in resilin-like polypeptides. Simulated single-chain *R*_g_ values at different temperatures for (A) ELPs and (B) RLPs. Experimentally observed outliers are represented by black solid lines and indicated by arrows. Outliers 1–7 exhibit no phase separation within the temperature range from 20 °C to 80 °C in experiments. Outlier 8 is an RLP-like sequence but undergoes LCST-type phase separation. The simulations correctly capture these trends. The normalized *R*_g_ values at 350 K for ELP sequences show a positive correlation with the experimentally measured cloud temperature *T*_cloud_ (C), while those for RLP sequences exhibit a negative correlation (D). *r*_U_ and *r*_L_ are the upper and lower bounds of the correlation coefficients for a 95% confidence interval. For better visualization of the identified outliers, Δ*R*_g_ = *R*_g_(350 K) – *R*_g_(300 K) was calculated for ELPs (E) and RLPs (F).

Remarkably, the TEA framework successfully identified outlier sequences reported in the experimental study by Quiroz and Chilkoti.^56^ The RLP sequences (VRPVG)_25_, (TVPGAG)_55_, (VAPGVG)_20_, (APGVG)_45_, and ELP sequences (VPSDDYGVG)_29_, (VPSDDYGQG)_20_, (VPHSRNGG)_40_, which are labeled as Exp. Outlier 1–7 in Figure 5A–B, do not undergo phase separation within the experimental temperature range from 20 °C to 80 °C. Our simulations show that these sequences, especially Exp. Outlier 1, 5, 6, exhibit extended conformations with high *R*_g_ values at all the simulated temperatures from 250 to 400 K, indicating the absence of phase separation. Importantly, our simulations show that the RLP sequence Exp. Outlier 8 (VPSTDYGVG)_12_ exhibits LCST-type single-chain coil-to-globule transitions, which agrees with its LCST-type phase behavior observed in experiments. To clarify the identification of experimental outliers, we quantified the thermoresponsive trend by calculating the change in dimensions, Δ*R*_g_= *R*_g_(350 K) – *R*_g_(300 K). UCST sequences expand upon heating (Δ*R*_g_> 0), while LCST sequences collapse (Δ*R*_g_< 0). As shown in Figure 5E and F, our TEA-augmented model correctly predicts the inverted sign for several experimental outliers, successfully identifying sequences whose phase behavior contradicts standard heuristic expectations.

It is important to note that the correlation between single-chain dimensions and macroscopic phase behavior can break down depending on specific sequence features, such as net charge per residue, as highlighted by recent studies.^64,65^ In our data set, the majority of the ELP and RLP sequences have a net charge of zero, suggesting that deviations from linearity are likely driven by multichain cooperativity rather than electrostatics. However, five of the eight experimental outliers possess a nonzero net charge. The electrostatic repulsion in these highly charged sequences likely hinders their ability to phase-separate.

Further validation was performed on five IDPs previously studied by Wuttke et al., including ProTα N, ProTα C, Integrase, Csp Tm, and λ-repressor.^37^ It has been shown that the single-chain *R*_g_ values of these IDPs decrease with increasing temperature due to temperature-dependent solvation. The TEA-augmented HPS-Urry and CALVADOS (M2) models successfully reproduced the temperature-dependent *R*_g_ values for Integrase and Csp Tm (Figures 6A–B and S15–S17). While the absolute *R*_g_ values for Integrase and Csp Tm from the TEA-augmented HPS-FB model were underestimated, the degree to which they change with temperature matched the experimental trend (Figure S16). For λ-repressor, all three models overestimated the *R*_g_ values but resulted in the LCST-type single-chain coil-to-globule transition curves (Figures 6C, S15–S17). Given that our current implementation of the TEA approach is specifically to fit the temperature dependence of λ values, we believe that its performance in capturing the temperature-dependent single-chain behavior is reasonable and acceptable. Further improvement can be achieved by adjusting λ values at the reference temperature in the original CG models, which is beyond the scope of this study. For the highly charged ProTα N and ProTα C, simulated *R*_g_ values from all three models show negligible change with temperature (Figure 6D–E, S15–S17). This inconsistency with experimental results is likely due to the absence of explicit ion effects in these implicit solvent CG models. The simulated *R*_g_ values at 350 K of these five sequences also show strong correlations with experimental results (Figures 6F, S15–S17), further demonstrating the ability of the TEA approach in capturing the temperature-dependent single-chain behaviors. It is worth noting that Wuttke et al. provided the single-molecule FRET data instead of directly measuring *R*_g_ values.^37^ Similar to the work of Dignon et al.,^41^ we converted the single-molecule FRET data using the SAW-ν model,^66^ which may introduce uncertainties of the inferred *R*_g_ values. Taken together, these results demonstrate that the TEA approach can successfully capture the temperature-dependent single-chain behaviors of IDPs, although it shows limitations on highly charged sequences due to the inherent limitations of the coarse-grained models.

**Figure 6:**
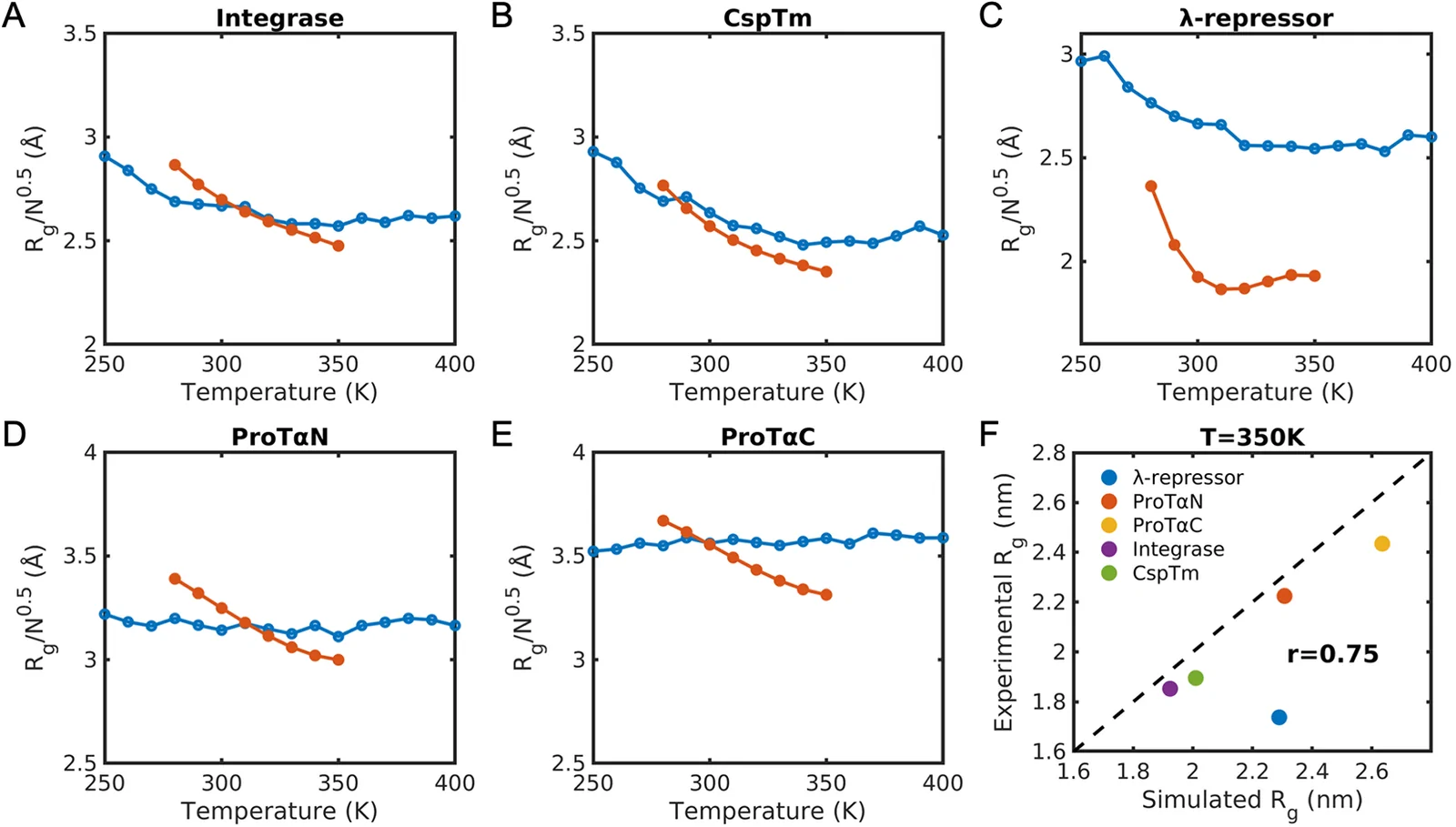
Comparison of simulated and experimental single-chain dimensions for five IDPs exhibiting LCST-type phase behavior. Temperature-dependent *R*_g_ values from the TEA-augmented HPS-Urry model with γ = 2 (blue curves) are compared with those inferred from single-molecule FRET data from Wuttke et al.^37^ (red curves) for (A) Integrase, (B) CspTm, (C) λ-repressor, (D) ProTαN, and (E) ProTαC. (F) Correlation between simulated and experimental *R*_g_ values at 350 K. The single-molecule FRET data were converted to *R*_g_ values using the SAW-ν model.^66^

To systematically calibrate the scaling factor γ, we evaluated values ranging from 1 to 4 for the TEA-augmented HPS-Urry model. We calculated the mean absolute error (MAE) against single-chain dimensions from Wuttke et al., as well as the Pearson correlation coefficients between simulated *R*_g_ values and experimental cloud point temperatures for ELP and RLP sequences (Figure S18 and Tables S5 and S6). We found that γ = 2 for the TEA-augmented HPS-Urry model offers the most balanced performance, maintaining a low MAE for single-chain dimensions while robustly capturing the distinct thermoresponsive trends of both LCST and UCST sequences.

To validate the model’s ability to capture diverse phase behaviors, we computed phase diagrams for IDPs with varying thermoresponsive properties using the TEA-augmented HPS-Urry model. We selected three ELP and RLP sequences for analysis. As illustrated in Figure 7A–B, the simulations correctly predict LCST-type phase separation for the ELP sequences and UCST-type behavior for the RLP sequences.

**Figure 7:**
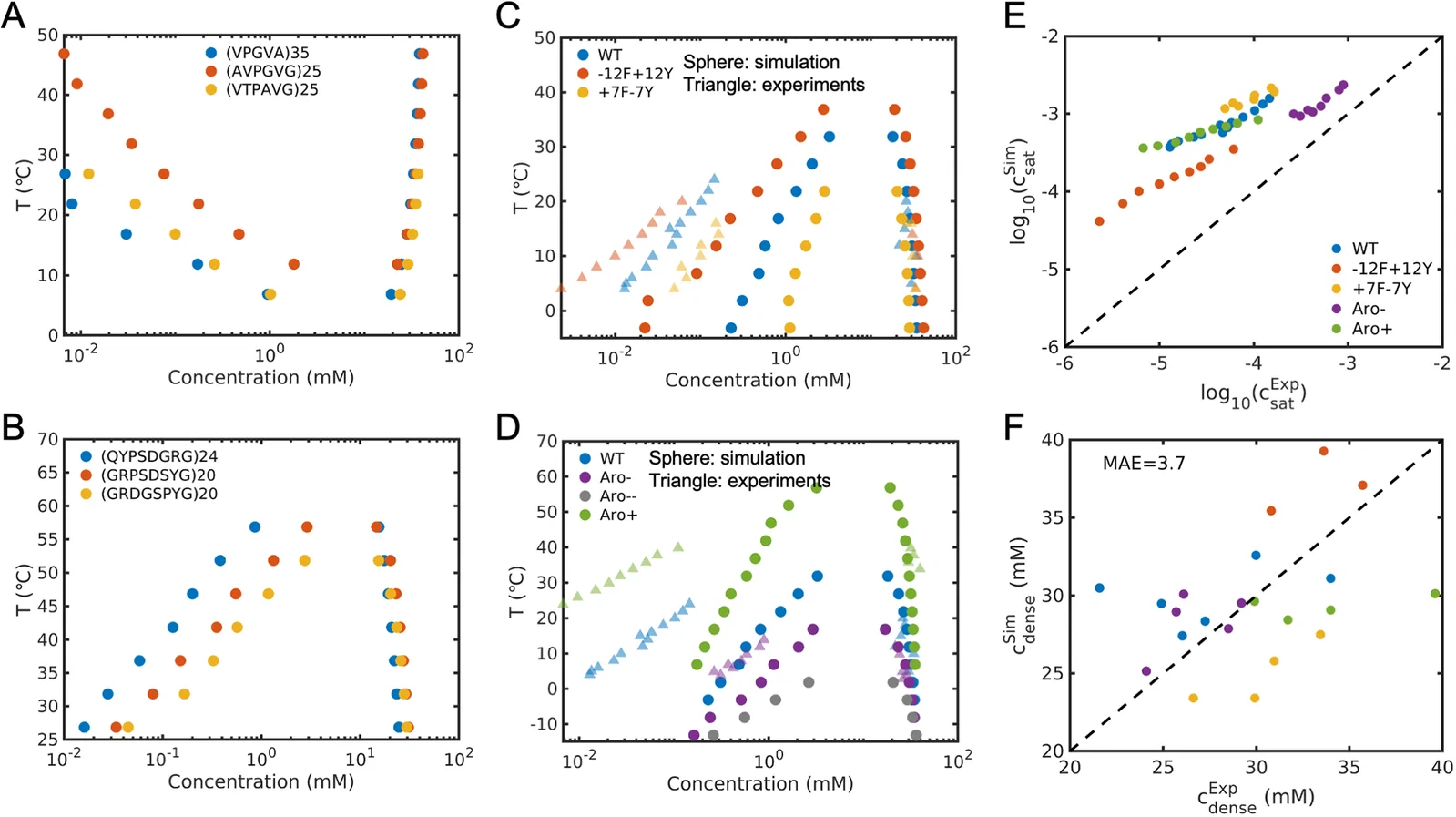
TEA-augmented HPS-Urry model reproduces phase diagrams for diverse IDP sequences: Phase diagrams for (A) three ELP sequences, (B) three RLP sequences, (C) A1-LCD and two variants with altered numbers of Tyr and Phe, and (D) A1-LCD and three variants with different patterning of aromatic residues. Comparison between the simulated versus experimental (E) saturation concentration (*c*_sat_) and (F) protein concentration in the dense phase (*c*_dense_). The ELP and RLP sequences are selected from Quiroz and Chilkoti.^56^ Experimental data indicated by triangles in (C) and (D) for A1-LCD and its variants are from the work of Mittag’s group.^63,64^

We extended this analysis to A1-LCD and its variants, which are known to exhibit UCST-type phase behavior.^63,64^ As shown in Figure 7C, the model successfully reproduces the experimental phase boundaries. Previous studies have established that tyrosine (Tyr/Y) acts as a stronger “sticker” for phase separation than phenylalanine (Phe/F).^7,64,67^ Consequently, mutating Tyr to Phe increases the saturation concentration *c*_sat_ $c_{sat}$ . The TEA-augmented HPS-Urry model correctly captures the relative magnitudes of these shifts: the Tyr-enriched variant (−12F+12Y) exhibits a lower *c*_sat_, while the Phe-enriched variant (+7F–7Y) exhibits a higher *c*_sat_ compared to the wild type (WT). Finally, consistent with experimental findings, the model demonstrates that sequences with higher aromatic content possess a stronger propensity for phase separation and lower saturation concentrations (Figure 7D).

To quantitatively assess the accuracy of the phase diagrams, we directly compared the simulated dilute phase (*c*_sat_) and dense phase (*c*_dense_) concentrations with experimental data for A1-LCD and its variants (Figure 7E–F). While the simulated *c*_sat_values correctly capture the relative rank-order and temperature-dependent trends of the variants, their absolute *c*_sat_ values overestimate the experimental concentrations. This overestimation is a limitation of the HPS-Urry parametrization. We noted that although CALVADOS (M2) and mpipi models exhibit a similar issue, updated models CALVADOS 2 ^68^ and mpipi-Recharged^47^ can reproduce *c*_sat_ values with high quantitative accuracy.^69^ Conversely, the simulated *c*_dense_ values show remarkable quantitative agreement with the experimental measurements, demonstrating the model’s capability to accurately capture the density of the condensed phase.

We further compared the phase diagrams obtained from TEA-augmented HPS-Urry with those from standard HPS-Urry with temperature-independent interactions for A1-LCD and its variants (Figure S19). Since the TEA-augmented HPS-Urry retains the original λ values at the reference temperature of 300 K, the dilute and dense phase concentrations show minimal deviation around 300 K for all six sequences. The phase diagrams for the −12F+12Y variant are nearly identical between the two models, whereas the +7F–7Y variant exhibits a more significant deviation. This difference can be attributed to the stronger temperature dependence of Phe than Tyr as shown in Figure 1. For the Aro- and Aro– variants, the dilute phase arms show mild variations while preserving the overall shape of the binodal. In contrast, the phase diagram for Aro+ displays a larger deviation and exhibits a crossover at the reference temperature, which is likely driven by the strong cooperativity between aromatic residues due to their increased linear clustering. Overall, the TEA-augmented HPS-Urry model preserves the ability to capture phase diagrams for UCST-type sequences while simultaneously enabling the description of LCST-type phase behavior.

## Discussion

In this study, we introduced the TEA framework to incorporate temperature-dependent interactions into CG models for simulating thermoresponsive behaviors of IDPs. By employing extensive all-atom MD simulations, we successfully obtained the temperature-dependent energetics of amino acid side chain analogues and further converted them to a coarse-grained potential. When augmented with TEA, coarse-grained models effectively distinguish between sequences that undergo LCST-type phase separation and UCST-type phase separation. Notably, this framework successfully identifies experimental outliers and captures both LCST-type and UCST-type phase behaviors, demonstrating its significant predictive power.

### A Physics-Driven Approach for Transferability

Unlike a purely data-driven approach that relies on fitting large experimental data sets, the TEA approach is fundamentally physics-based. It includes two stages: (a) quantify the temperature-dependent energetics from extensive all-atom MD simulations; (b) map this atomistic energetics to CG potentials via a single free parameter γ. This approach offers two advantages. First, this one-parameter fitting process minimizes the risk of overfitting when high-quality data is scarce. Second, the physics-based approach enhances the model’s interpretability and transferability, allowing it to predict behaviors outside the training set while providing a clear understanding of its physical boundaries.

A key step of our approach is connecting the inter-residue interaction strength quantified by excess free energies Δ*G*_E_ with hydration free energies Δ*u*_h_. We establish a linear relationship between Δ*G*_E_ and Δ*u*_h_ by a residue-specific scaling factor κ_*i*_ as shown in eq 3. The linearity is well supported by the all-atom simulations (Figure 3). Furthermore, we employed a combination rule to derive heterotypic interactions from homotypic data. The high correlation between simulated heterotypic interactions and those predicted by the combination rule confirms that the combination rule works well for these rigid side chain analogues (Figure 4).

In addition to the arithmetic mean, we also investigated whether heterotypic Δ*G*_AB_ values could be approximated using the geometric average of the corresponding homotypic interactions (Δ*G*_AA_ and Δ*G*_BB_). While calculating the geometric mean is straightforward for homotypic pairs sharing the same sign, pairs with opposite signs require a mathematical adjustment. To account for this, we applied a sign-adjusted geometric mean, where the sign of the predicted heterotypic interaction is determined by the sum of the homotypic values $\left(\Delta G_{AB}=\mathrm{sign}\left(\Delta G_{AA}+\Delta G_{BB}\right)\sqrt{\left|\Delta G_{AA}\Delta G_{BB}\right|}\right)$ . Our analysis revealed that this geometric average approach also yields robust approximations of heterotypic interactions, demonstrating comparable Pearson correlation coefficients and mean absolute errors to those obtained using arithmetic averages (Figure S20). Ultimately, we chose to highlight the arithmetic average in our primary analysis because it provides a simpler, more universally applicable combination rule that intrinsically handles opposite-sign interactions without requiring conditional adjustments.

### Fit Lambda Values from All-Atom Energetics

We directly set the λ values at the reference temperature, λ­(*T*_0_), to be the original λ values in the HPS-based models and obtain the temperature dependence of λ values from Δ*G*_E_ using a linear relationship (eq 6). By combining eqs 2, 3, and 6, λ becomes an analytical function of thermodynamic observables Δ*u*_h_(*T*_0_), Δ*c*_p_, Δ*h*, the scaling factor κ, and the fitting parameter γ. While the thermodynamic terms and κ are obtained directly from the all-atom simulation, γ is the sole tunable parameter obtained to match experimental data. This approach simplifies the fitting process significantly. We note that λ­(*T*_0_) can be derived from Δ*G*_E_ (*T*_0_) in principle, which will introduce additional fitting parameters besides γ. Therefore, we leverage existing CG models to constrain the reference values, thereby maintaining a simple but efficient model with minimal free parameters.

We point out that the influence of the coarse-grained bead radius σ on the interaction strength is ignored in parametrizing λ. A more rigorous alternative is to fit the λ values by connecting the all-atom Δ*G*_E_with the coarse-grained one $\Delta G_{E}^{cg}$ by a linear relationship. However, because $\Delta G_{E}^{cg}$ is not an elementary function of λ and depends on the residue-specific bead radius, such an approach makes the fitting process not straightforward and requires numerical solutions. Our simplified method maintains tractability while effectively capturing the essential physics. It is important to note that while we fit the temperature-dependent potential for the one-bead-per-residue models, the underlying energetics are derived from side chain analogues. Consequently, this implementation assumes that the backbone contribution is uniform across residues and that backbone-side chain cooperativity is negligible. Future refinements could employ two-bead-per-residue models to explicitly decouple backbone and side chain energetics.

Regarding the force field dependence, our current κ_*i*_ and Δ*u*_h_ values are derived using the CHARMM force field. We believe that κ_*i*_ is an intrinsic thermodynamic quantity and therefore the choice of force fields will not significantly influence the κ_*i*_ values, or alter the relative ranking of κ_*i*_ values. Moreover, experimental results of temperature-dependent hydration free energies are available for these 19 side chain analogues,^49,70–72^ and temperature-dependent second virial coefficients can be measured in experiments to calibrate λ values.^58,73,74^ Future iterations of TEA could incorporate this experimental data directly, which will render the framework force-field independent, potentially increasing its reliability.

### Robustness and Limitations

The TEA framework is highly versatile and compatible with existing one-bead-per-residue models. In this study, we successfully incorporated the temperature-dependent energetics into the HPS-Urry, HPS-FB, and CALVADOS (M2) coarse-grained models. Although the single-chain *R*_g_ values at 300 K differ for these models due to their intrinsic parametrization, the trend of the temperature-dependent single-chain behavior remains consistent across these three models. This suggests that the derived temperature-dependent energetics are robust and can be transferred. We anticipate that the TEA framework can be extended to other coarse-grained models for modeling biomolecular phase separation, such as mpipi,^28^ AWSEM-IDP,^75^ COCOMO,^76^ MOFF,^77^ and HyRes.^78^

However, we do not expect that a coarse-grained model can capture the thermoresponsive behavior of all types of IDPs if the necessary molecular features are ignored in the design of the coarse-grained model. Indeed, we found that the current TEA implementation does not accurately model highly charged IDPs, where ion effects are crucial. This is an expected consequence of using implicit solvent models that lack explicit ions. It is widely recognized that ion binding and releasing are important driving forces for polymer phase separation,^79–82^ and recent studies have demonstrated the necessity of explicit ions for simulating phase separation of highly charged biomolecules.^83–87^ Other critical factors are currently omitted from the model, including backbone-side chain cooperativity and many-body effects.^88^ Additionally, the use of a modified Lennard-Jones potential to represent solvent-mediated interactions neglects the desolvation barrier, which has recently been shown to play a vital role in regulating biomolecular phase separation.^89^ Addressing these missing factors will be an important direction for the future development of coarse-grained models.

## Methods

### Details of All-Atom Molecular Dynamics Simulations

All simulations were performed using Gromacs 2024.2 package.^90^ The CHARMM General Force Field (CGenFF)^44–46^ were used for model compounds, with the exception of the arginine analogue. We compared the Lennard-Jones parameters (σ and ε) and partial charges against those from CHARMM36m protein force field^19^ (Figures S21–S24). While values from these two force fields are nearly identical for most analogues, we observed a significant difference in the partial charge of guanidinium nitrogen in arginine analogue. Consequently, we adopted CHARMM36m parameters for arginine analogue to ensure consistency with protein parametrization. CHARMM modified TIP3P water model was used.^91^ All simulations were first energy minimized using the steepest descent algorithm. This was followed by an equilibration phase of 0.5 ns in the NPT ensemble. Production simulations were subsequently performed in the NPT ensemble. Temperature was kept at the target value using the v-rescale thermostat^92^ with a coupling time constant of 0.1 ps for the umbrella sampling, while the Langevin thermostat with an inverse friction constant of 2 ps was used for the hydration free energy calculations. Pressure was maintained at 1 bar by the Parrinello–Rahman barostat^93,94^ with a coupling constant of 2 ps. The distance cutoff for both short-range van der Waals and electrostatic interactions was 1.2 nm. Periodic boundary conditions were applied, and the Particle Mesh Ewald (PME) method^95,96^ was used to calculate the long-range electrostatic interactions. The integration time step is 2 fs. The integrators for umbrella sampling and hydration free energy calculations are *md* and *sd*, respectively.

### Umbrella Sampling for Getting Potential of Mean Forces (PMFs)

Umbrella sampling^97^ combined with the weighted histogram analysis method (WHAM)^98,99^ were used to obtain PMFs. For each system, nine windows with centers from 0.4 to 2.0 nm with an interval of 0.2 nm were used. In each window, 4 independent simulations were performed, each with 100 ns long. Therefore, the accumulated simulation time for the 19 homotypic interaction pairs at 6 different temperatures is 410.4 μs, and the accumulated simulation time for the 43 heterotypic interaction pairs at 300 K is 154.8 μs. The total simulation time for PMF calculations is 565.2 μs. The harmonic restraint potential with a force constant of 1.0 kcal·mol^–1^·Å^–2^ was applied to the distance between key functional groups in the model compounds. Key functional groups or atoms for each residue are listed in Table S4.

### Hydration Free Energy Calculations

Free energy perturbation simulations were performed, and the Bennett Acceptance Ratio (BAR) method^80^ implemented in GROMACS package was used to calculate the free energy difference between two adjacent λ windows. λ values for the van der Waals interactions and electrostatic interactions for each window are [λ_vdw_, λ_ele_] = [0, 0], [0.1, 0], [0.2, 0], [0.3, 0], [0.4, 0], [0.5, 0], [0.6, 0], [0.7, 0], [0.8, 0], [0.9, 0], [1.0, 0], [1.0, 0.1], [1.0, 0.2], [1.0, 0.3], [1.0, 0.4], [1.0, 0.5], [1.0, 0.6], [1.0, 0.7], [1.0, 0.8], [1.0, 0.9], [1.0, 1.0]. For each λ window, 10 ns simulations in the NPT ensemble were performed.

### Coarse-Grained Molecular Dynamics Simulations

The single-chain simulations were performed using the LAMMPS 2-Aug-2023 simulation package.^100^ For each sequence, a 2 μs long simulation for sequences with less than 200 residues and a 4 μs long simulation for longer sequences were performed to calculate the *R*_g_ values at each target temperature. The initial 50 ns was discarded as equilibration. A Langevin integrator with a time step of 10 fs and a friction coefficient of 0.5 ps^–1^ was used for the simulations. All interactions were truncated at 4 nm. The simulation box is 80 × 80 × 80 nm^3^. Temperature-dependent dielectric constants were used as in the CALVADOS^27^ and mpipi models.^28^ The inverse Debye screening length was set to 0.1 Å^–1^, corresponding to 0.1 M salt.

The multiple-chain simulations for calculating phase diagrams were performed using the CALVADOS package.^101^ The slab simulation box with a size of 15 × 15 × 150 nm^3^ was used. 100 proteins were inserted into the box with an overall concentration of 4.92 mM. For each temperature, 10 μs production simulations were performed. The first 1 μs was treated as equilibration, and the rest 9 μs was used to calculate the dilute and dense phase concentrations. CALVADOS package employs a CSV file (residues_CALVADOS2.csv) with residue-specific parameters as the input. We changed its lambda parameters within the file for each simulation temperature while keeping the sigma values unchanged. By employing HPS-Urry’s lambda values at 300 K and temperature-dependent lambda values at other temperatures, we can do the simulations with TEA-augmented HPS-Urry model. All interactions were truncated at 4 nm. The default values in CALVADOS package^101^ were used for all other parameters.

## Supplementary Material







## Notes

Supplementary tables and figures for thermodynamic parameters for hydration free energies of the 19 analogues, linear regression parameters between $\Delta G_{E}^{\prime }$ and Δ*u*_h_, categorization of 43 heterotypic interaction pairs, key functional groups in each analogue used to calculate the intermolecular distance for applying the harmonic potential in umbrella sampling, temperature-dependent potential of mean forces for the homotypic interactions of the 19 analogues, correlation between excess free energies at 300 K calculated from all-atom simulations, Δ*G*_E_ (300 K) and hydrophobicity scales in existing CG models, *G*_E_ versus Δ*u*_h_ for charged analogues, PMFs for heterotypic interactions at 300 K, performance of TEA-augmented CALVADOS (M2) and HPS-FB models, comparison of simulated and experimental single-chain dimensions for five IDPs exhibiting LCST-type phase behavior, impact of temperature-dependent energetics on phase diagrams, correlation between excess free energies calculated using the molecular-specific cutoff, $\Delta G_{E}^{\prime }$ , at 300 K and those derived using the 10 Å cutoff, correlation between excess free energies $\Delta G_{E}^{\prime }$ exhibit and hydration free energies Δ*u*_h_, comparison of the sigma, epsilon, and partial charges in CGenFF and CHARMM36m protein force fields for the analogues (PDF)
Raw data for Table 1 and Table S1 (CSV)
An example folder for running LAMMPS simulations with TEA-augmented coarse-grained models (ZIP)
