# Supplementary material for: Simulating Thermoresponsive Behavior of Disordered Proteins with Temperature-Dependent Coarse-Grained Potentials Derived from Hydration Free Energies
Source: JACS Au. 2026 Aug 3;6(8):4478–91. doi: 10.1021/jacsau.6c00523 (PMC13508075; doi:10.1021/jacsau.6c00523)
Supplement: Supplementary file 1 [file au6c00523_si_001.pdf]

Supplementary Information

for

Simulating Thermoresponsive Behavior of Disordered Proteins with Temperature-Dependent Coarse-Grained Potentials Derived from Hydration Free Energies

Fangke Chen [1] and Xiangze Zeng [1,2 *]

[1] Department of Physics, Hong Kong Baptist University, Kowloon Tong, Hong Kong, China

[2] The Computational Medicine Lab, School of Chinese Medicine, Hong Kong Baptist University, Kowloon Tong, Hong Kong, China

E-mail: xiangzezeng@hkbu.edu.hk

Table S1. Thermodynamic parameters for hydration free energies. Parameters are obtained by fitting the temperature-dependent hydration free energies for the 19 amino acid sidechain/backbone analogues to the integrated version of the Gibbs-Helmholtz equation.

**Residue/unit**	**Model compound**	$\Delta u_h(300K)$ **kcal/mol**	**Δh kcal/mol**	Δc_p **cal/molK**	**MAE kcal/mol**
Ala	Methane	2.45	0.02	35.30	0.006
Val/Pro	Propane	2.54	-1.13	40.50	0.010
Leu	2-methylpropane	2.90	-1.17	50.49	0.018
Ile	*n*-butane	2.74	-1.93	62.68	0.012
Met	Ethyl methyl thioether	1.03	-3.75	43.66	0.010
Phe	Toluene	0.37	-5.71	63.67	0.009
Cys	Methanethiol	-0.02	-3.69	39.10	0.009
Tyr	*p*-Cresol	-4.00	-10.60	41.91	0.012
Trp	3-methylindole	-5.25	-12.94	50.57	0.023
Ser	Methanol	-4.38	-8.83	21.47	0.009
Thr	Ethanol	-4.24	-10.10	37.74	0.007
Asn	Acetamide	-7.53	-13.01	24.54	0.004
Gln	Propionamide	-7.27	-13.80	36.41	0.008
His	4-methylimidazole	-10.55	-18.72	41.86	0.017
Backbone/Gly	*N*-methylacetamide	-7.15	-14.06	41.81	0.012
Arg	*n*-propylguanidine	-45.74	-57.99	58.02	0.014
Lys	1-butylamine	-53.54	-62.55	41.93	0.004
Asp	Acetic acid	-97.11	-111.95	3.49	0.020
Glu	Propionic acid	-97.05	-112.90	4.68	0.012

Table S2. Linear regression parameters between $\boldsymbol{\Delta G_E'}$ and $\boldsymbol{\Delta u_h}$. $\Delta G_E'$ is the excess free energy calculated using the molecular-specific cutoff $r_{cut,i}$. κ_i' is the slope and r_i' is the correlation coefficient for analogue *i*.

Residue/unit	κ_i'	r_i'	Mean absolute error (kcal/mol)	Cutoff $r_{cut,i}$ (Å)
Ala	-0.392	-0.943	0.019	6.0
Val/Pro	-0.260	-0.981	0.013	7.4
Leu	-0.333	-0.974	0.022	7.6
Ile	-0.264	-0.962	0.024	8.2
Met	-0.138	-0.997	0.005	9.6
Phe	-0.144	-0.996	0.005	8.6
Cys	-0.177	-0.974	0.009	6.4
Tyr	-0.055	-0.969	0.008	8.8
Trp	-0.031	-0.962	0.006	11.4
Ser	-0.002	-0.886	0.000	6.6
Thr	-0.013	-0.776	0.005	9.0
Asn	-0.012	-0.497	0.009	6.8
Gln	-0.021	-0.941	0.004	7.6
His	-0.002	-0.299	0.005	7.4
Backbone/Gly	-0.014	-0.862	0.005	7.8
Arg	-0.014	-0.940	0.005	9.8
Arg (charmm36m)	0.063	0.979	0.012	9.8
Lys	-0.008	-0.761	0.006	10.3
Asp	-0.001	-0.122	0.010	9.2
Glu	0.002	0.354	0.009	9.2

Table S3. Categorization of 43 heterotypic interaction pairs. PMFs at 300 K for these pairs are calculated from all-atom molecular simulations. The one-letter amino acid code was used to represent the corresponding analogue.

Type	Heterotypic pairs
Hydrophilic-Hydrophilic	G-N; G-Q; G-S; G-T; G-H; G-C
Hydrophobic-Hydrophilic	S-T; Y-N; Y-Q; Y-S; Y-T; G-A; G-M; G-Y; G-V; G-L; G-I; S-A; T-A; G-W; G-F; Y-H; Y-C
Hydrophobic-Hydrophobic	Y-A; Y-M; Y-V; Y-L; Y-I; M-L; M-I; L-I; Y-W; Y-F
Cation-π	R-Y; R-W; R-F; K-Y; K-W; K-F
Charge-Charge	R-D; R-E; K-D; K-E

Table S4. Key functional groups in each analogue used to calculate the inter-molecular distance for applying the harmonic potential in umbrella sampling.

Residue/unit	Model compound	Functional group or atom
Ala	Methane	Carbon atom
Val/Pro	Propane	Carbon atom in the middle
Leu	2-methylpropane	Carbon atom at the center
Ile	*n*-butane	Carbon atom in the middle
Met	Ethyl methyl thioether	Sulfur atom
Phe	Toluene	Aromatic ring
Cys	Methanethiol	Sulfur atom
Tyr	*p*-Cresol	Aromatic ring
Trp	3-methylindole	Aromatic ring
Ser	Methanol	Oxygen atom
Thr	Ethanol	Oxygen atom
Asn	Acetamide	Carbon atom bonded to oxygen
Gln	Propionamide	Carbon atom two bonds away from oxygen
His	4-methylimidazole	5-membered ring
Backbone/Gly	*N*-methylacetamide	Carbon atom bonded to oxygen
Arg	*n*-propylguanidine	Carbon atom in guanidinium group
Lys	1-butylamine	Nitrogen atom
Asp	Acetic acid	Carbon atom bonded to oxygen
Glu	Propionic acid	Carbon atom bonded to oxygen

Table S5. The mean absolute error (MAE) against single-chain dimensions from Wuttke et al. The black lines represent the average MAE values for the five proteins.

	λ-repressor	ProTαN	ProTαC	Integrase	CspTm	Average
URRY, γ=1	0.673	0.120	0.136	0.107	0.152	0.238
URRY, γ=2	0.641	0.101	0.131	0.066	0.090	0.206
URRY, γ=3	0.577	0.104	0.142	0.034	0.040	0.179
URRY, γ=4	0.560	0.094	0.138	0.058	0.069	0.184
M2, γ=2	0.802	0.139	0.150	0.105	0.133	0.266
M2, γ=3	0.777	0.126	0.151	0.084	0.095	0.247
FB, γ=2	0.387	0.190	0.124	0.123	0.331	0.231
FB, γ=3	0.345	0.204	0.114	0.158	0.375	0.239

Table S6. Pearson correlation coefficients between simulated R_g values and experimental cloud point temperatures for ELP and RLP sequences. r_U and r_L are the upper and lower bounds for a 95% confidence interval, calculated using the *corrcoef* function in Matlab.

	LCST Sequences			UCST Sequences		
	r	r_L	r_U	r	r_L	r_U
URRY, γ=1	0.72	0.39	0.88	-0.65	-0.89	-0.13
URRY, γ=2	0.7	0.36	0.88	-0.7	-0.91	-0.21
URRY, γ=3	0.67	0.32	0.86	-0.74	-0.92	-0.3
URRY, γ=4	0.64	0.27	0.85	-0.77	-0.93	-0.35
M2, γ=2	0.33	-0.15	0.68	-0.84	-0.95	-0.51
M2, γ=3	0.47	0.03	0.76	-0.83	-0.95	-0.5
FB, γ=2	0.36	-0.12	0.7	-0.94	-0.98	-0.78
FB, γ=3	0.41	-0.05	0.73	-0.91	-0.98	-0.71

Table S7. Protein sequences from Wuttke et al.

λ-repressor	GPCLTQEQLEDARRLKAIYEKKKNELGLSQESVADKMGMGQSGVGALFNGINALNAYNA ALLAKILKVSVEEFSPSIARECR
ProTαN	CDAAVDTSSEITTKDLKEKKEVVEEAENGRDAPANGNAENEENGEQEADNEVCEEC
ProTαC	CEEGGEEEEEEEEGDGEEEDGDEDEEAESATGKRAAEDDEDDDVDTKKQKTDEDC
Integrase	CFLDGIDKAQEEHEKYHSNWRAMASDFNLPPVVAKGIVASCDKCQLKGEAMHGQVDC
CspTm	MRGKVKWFDSKKGYGFITKDEGGDVFVHWSAIEMEGFKTLKEGQVVEFEIQEGKKGPQA AHVKVVE

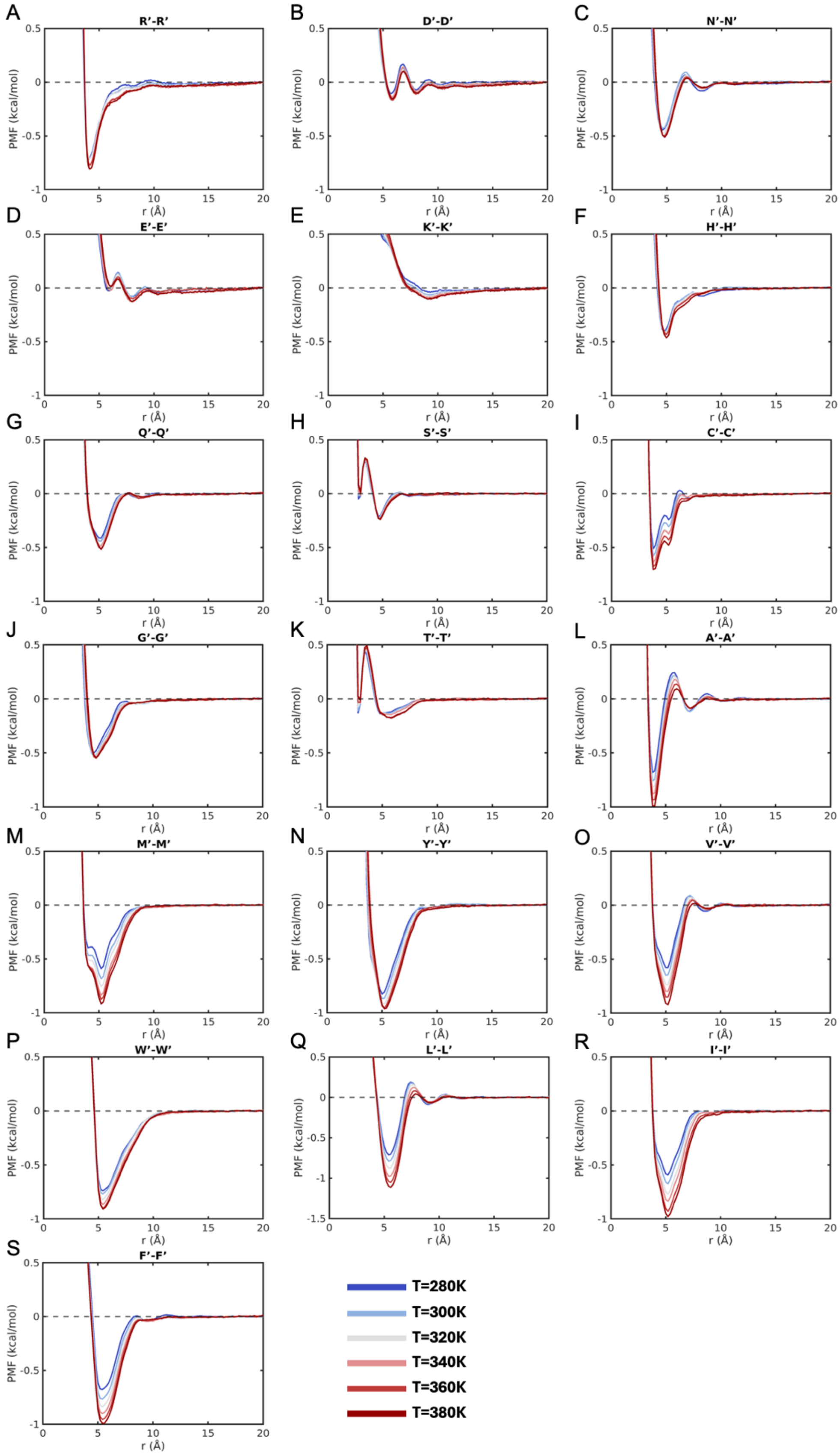

Figure S1. Temperature-dependent potential of mean forces for the homotypic interactions of the 19 analogues.

A

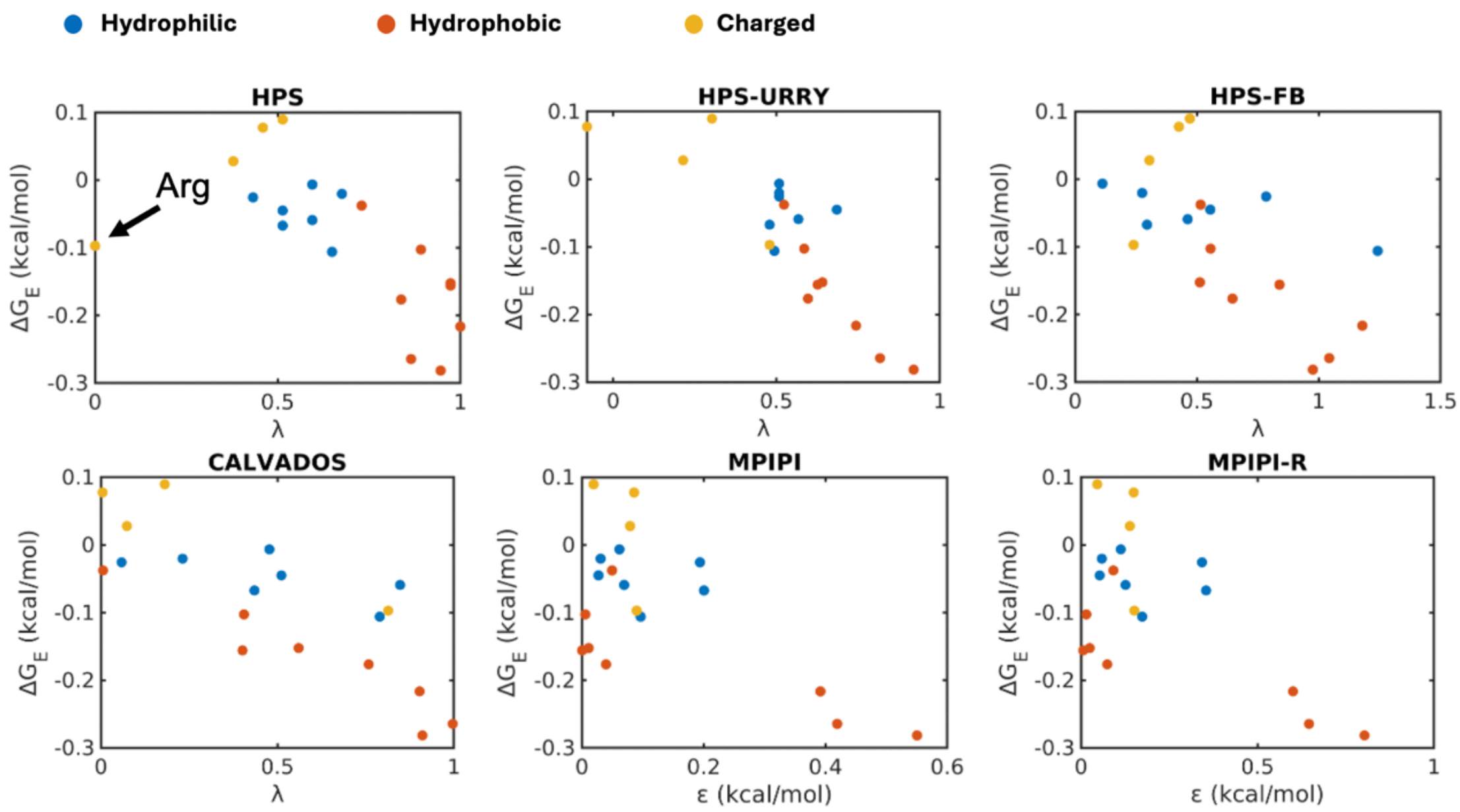

B

	HPS	HPS-URRY	HPS-FB	CALVADOS	MPIPI	MPIPI-R
**All analogues**	0.634	0.849	0.654	0.792	0.638	0.615
**Without Charged**	0.777	0.850	0.690	0.733	0.689	0.659

C

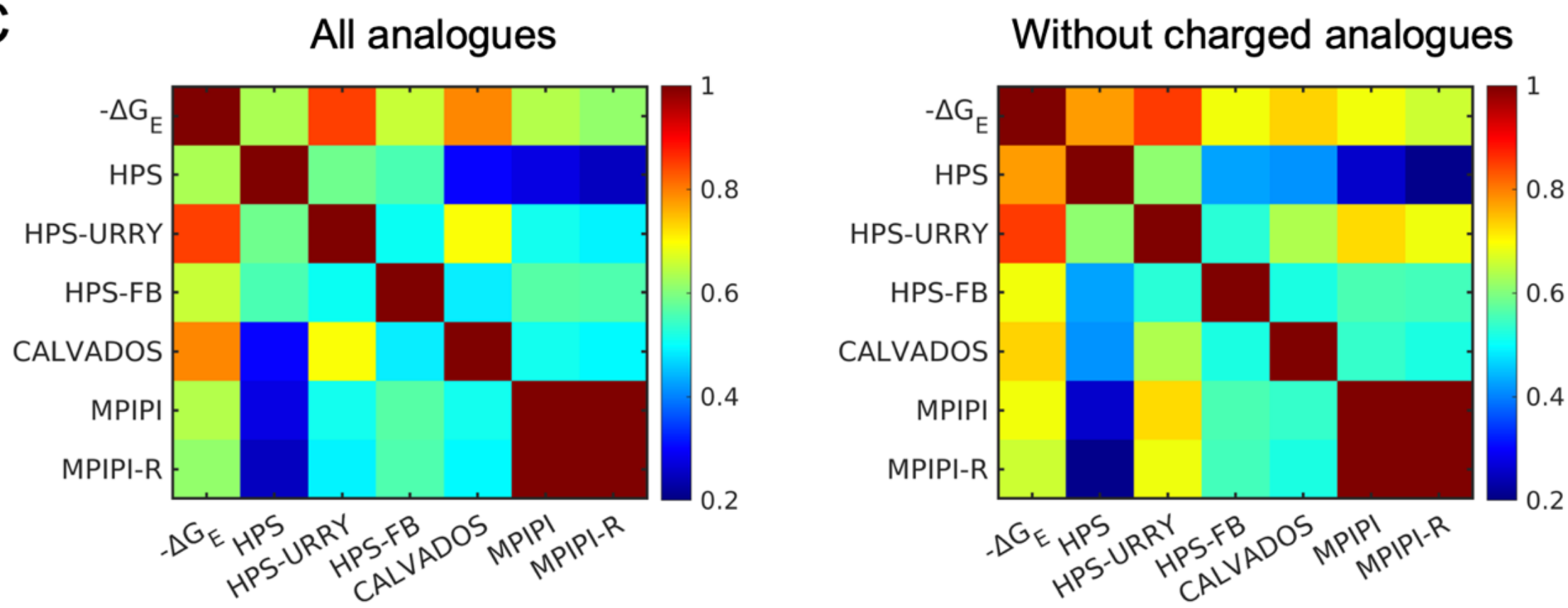

Figure S2. Excess free energies at 300 K calculated from all-atom simulations, $\Delta G_E(300K)$, exhibit strong correlation with hydrophobicity scales in existing CG models. (A) Scatter plot comparing $\Delta G_E(300K)$ against hydrophobicity scales in HPS, HPS-Urry, HPS-FB, CALVADOS, mpipi, and mpipi-R CG models, and (B) the corresponding correlation coefficients. (C) The cross correlation coefficient matrix across $\Delta G_E(300K)$ and hydrophobicity scales in these CG models.

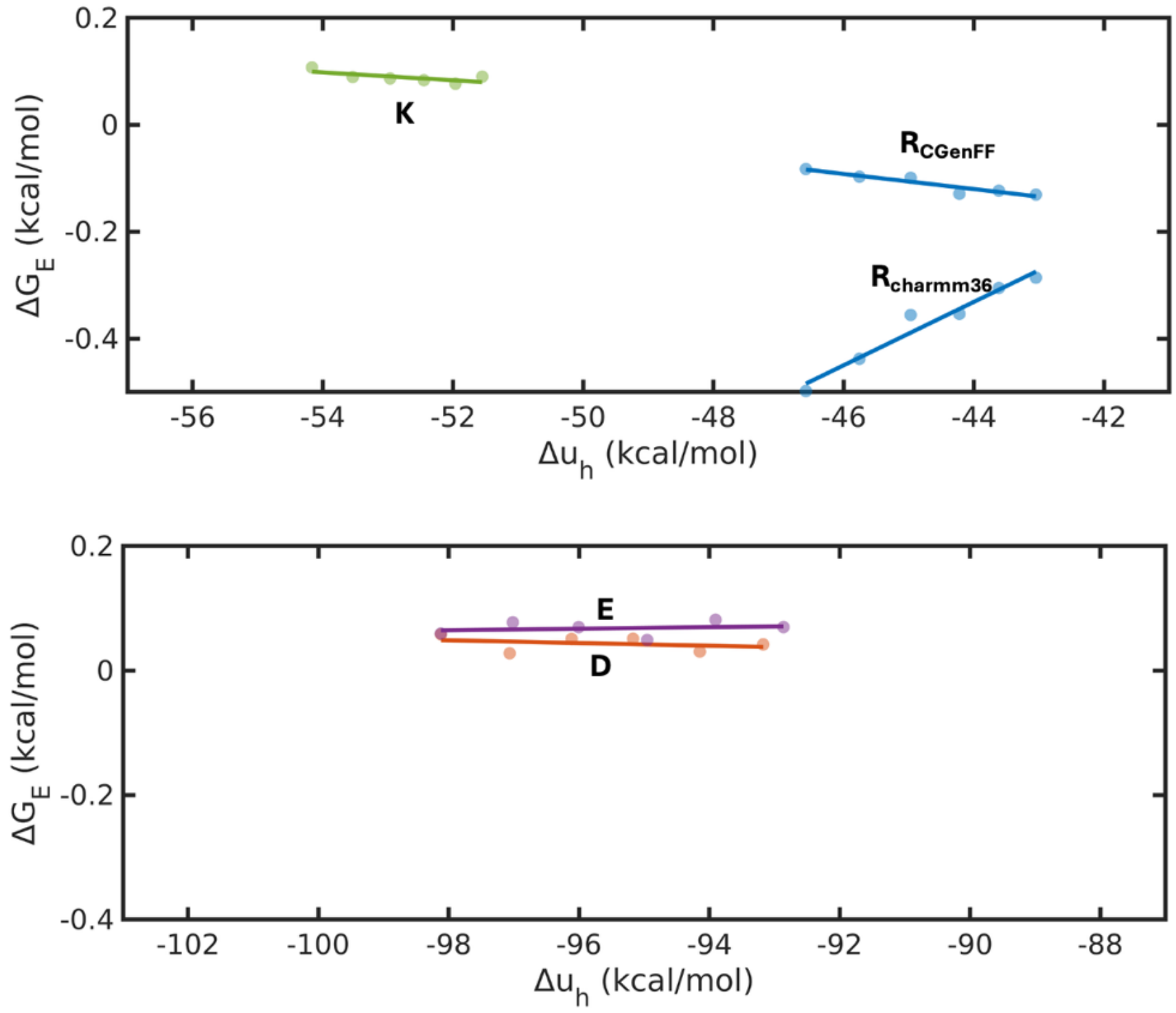

Figure S3. ΔG_E versus Δu_h for charged analogues. Data for K, E, D, R_{CGenFF} are from all-atom simulations using CGenFF, while those for $R_{charmm36}$ are from Charmm36m protein force field.

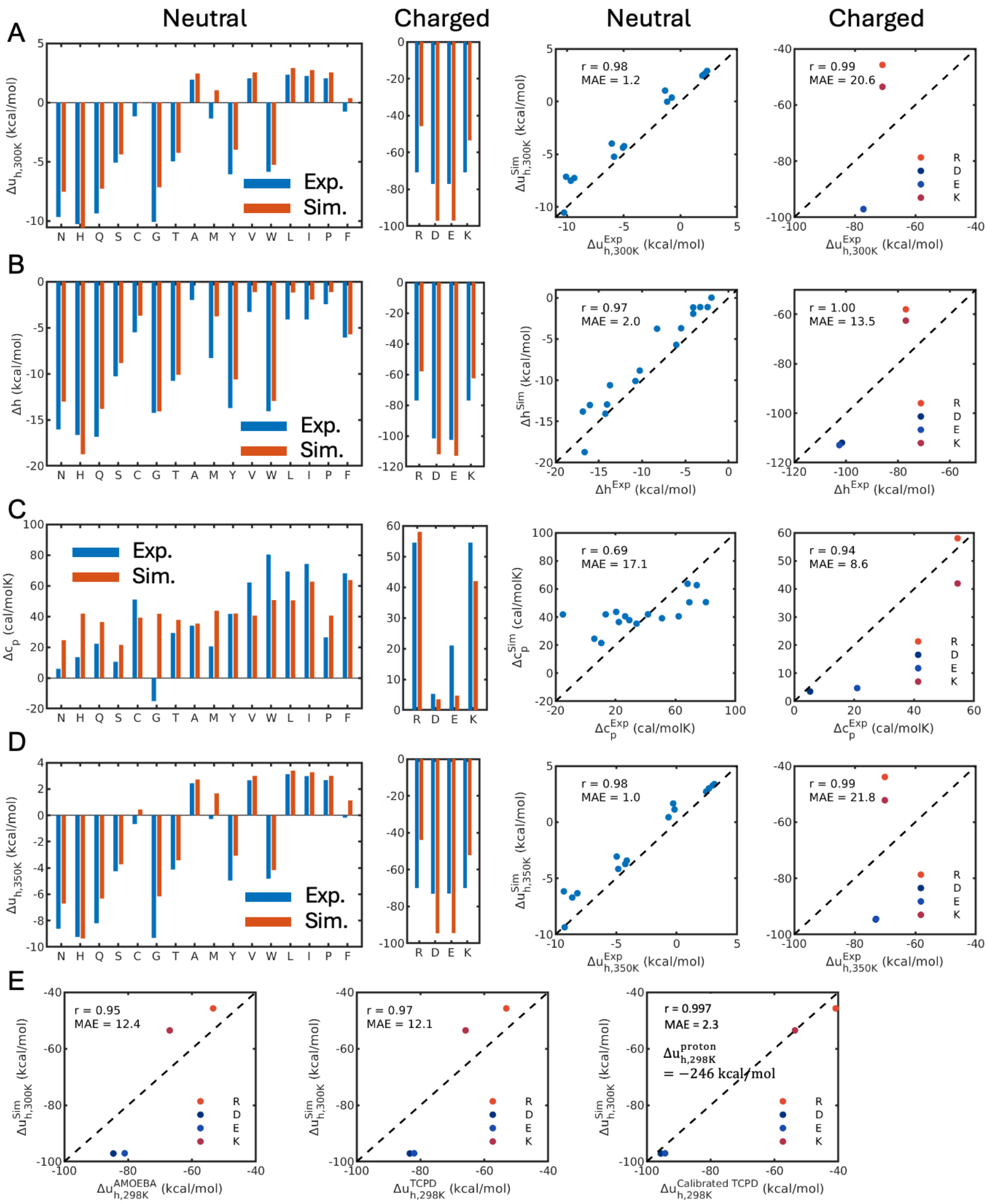

Figure S4. Comparison between the simulated thermodynamic parameters ($\mu_h(T_0), \Delta h, \Delta c_p$) with experimental values: (A) hydration free energies at 300 K, $\mu_h(300K)$, (B) Δh, (C) Δc_p, and (D) hydration free energies at 350 K. Experimental values for $\mu_h(300K)$ are from the ABSINTH model (Vitalis and Pappu, JCC, 2008) used by Wuttke et al. Experimental values for Δh and Δc_p are from Makhatadze and Privalov (JMB 1993).

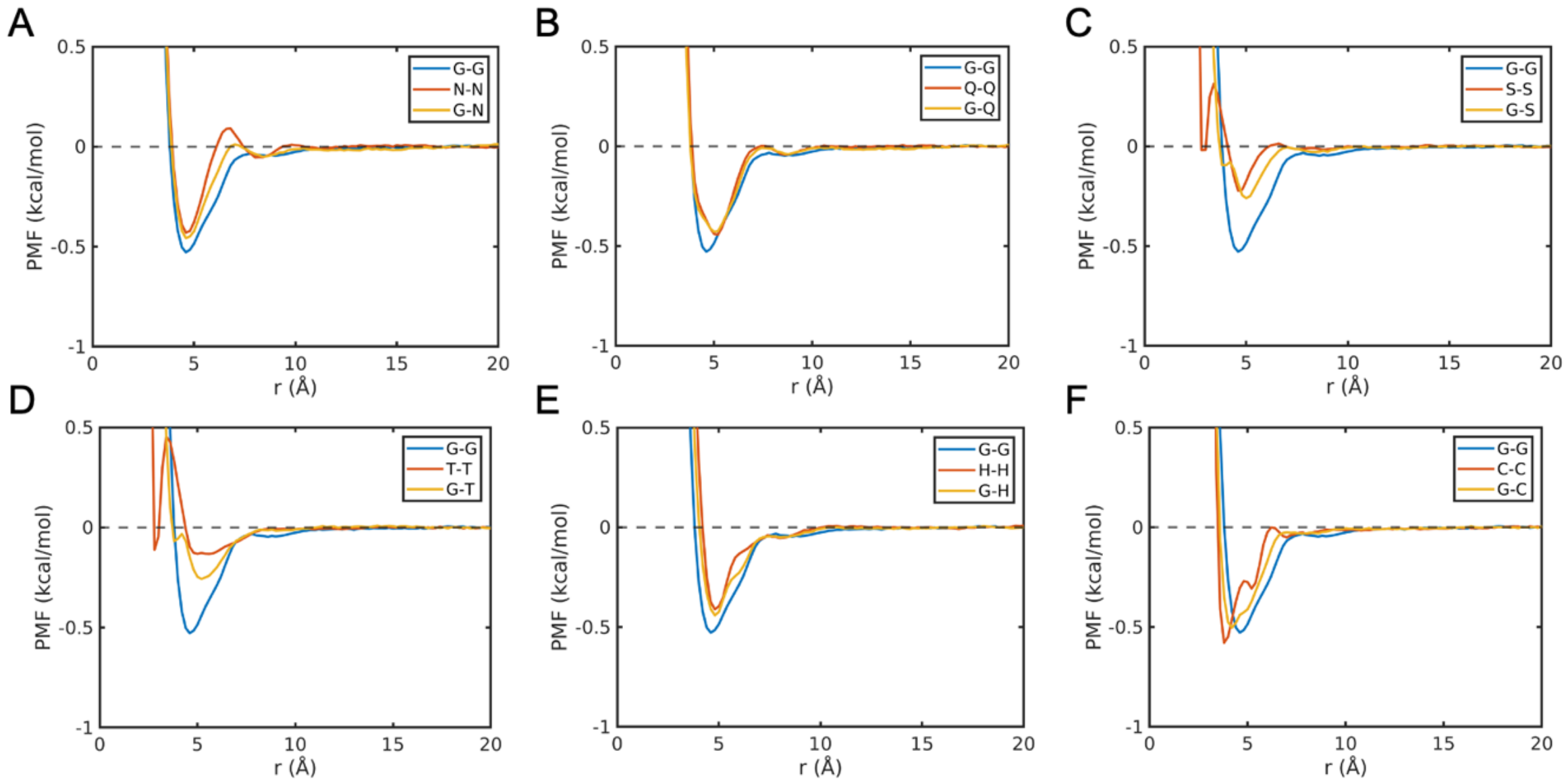

Figure S5. PMFs for the 'hydrophilic-hydrophilic' heterotypic interactions at 300 K. Heterotypic interactions are shown by yellow curves. For comparison, the corresponding homotypic interactions are shown in blue and red.

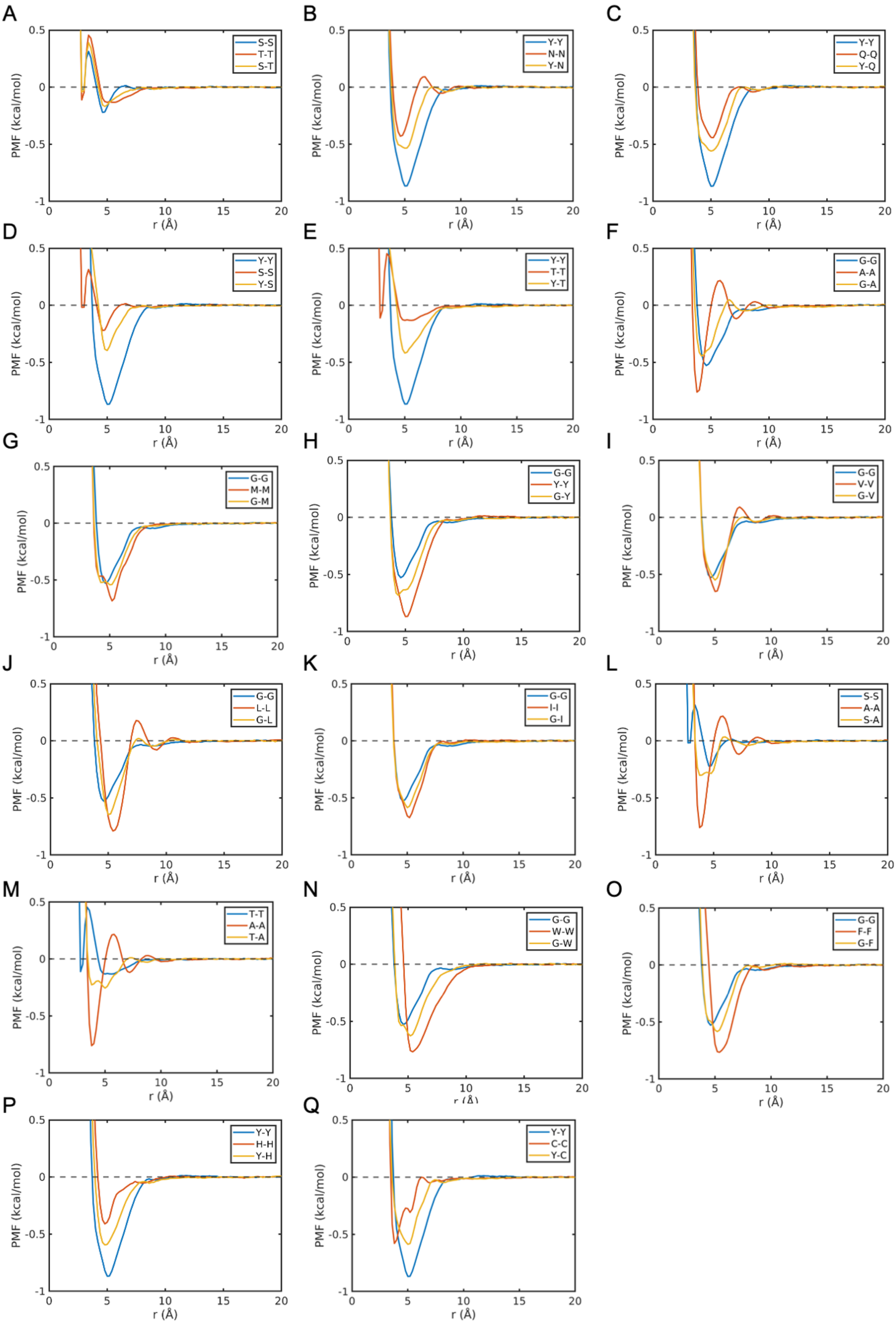

Figure S6. PMFs for the 'hydrophobic-hydrophilic' heterotypic interactions at 300 K. Heterotypic interactions are shown by yellow curves. For comparison, the corresponding homotypic interactions are shown in blue and red.

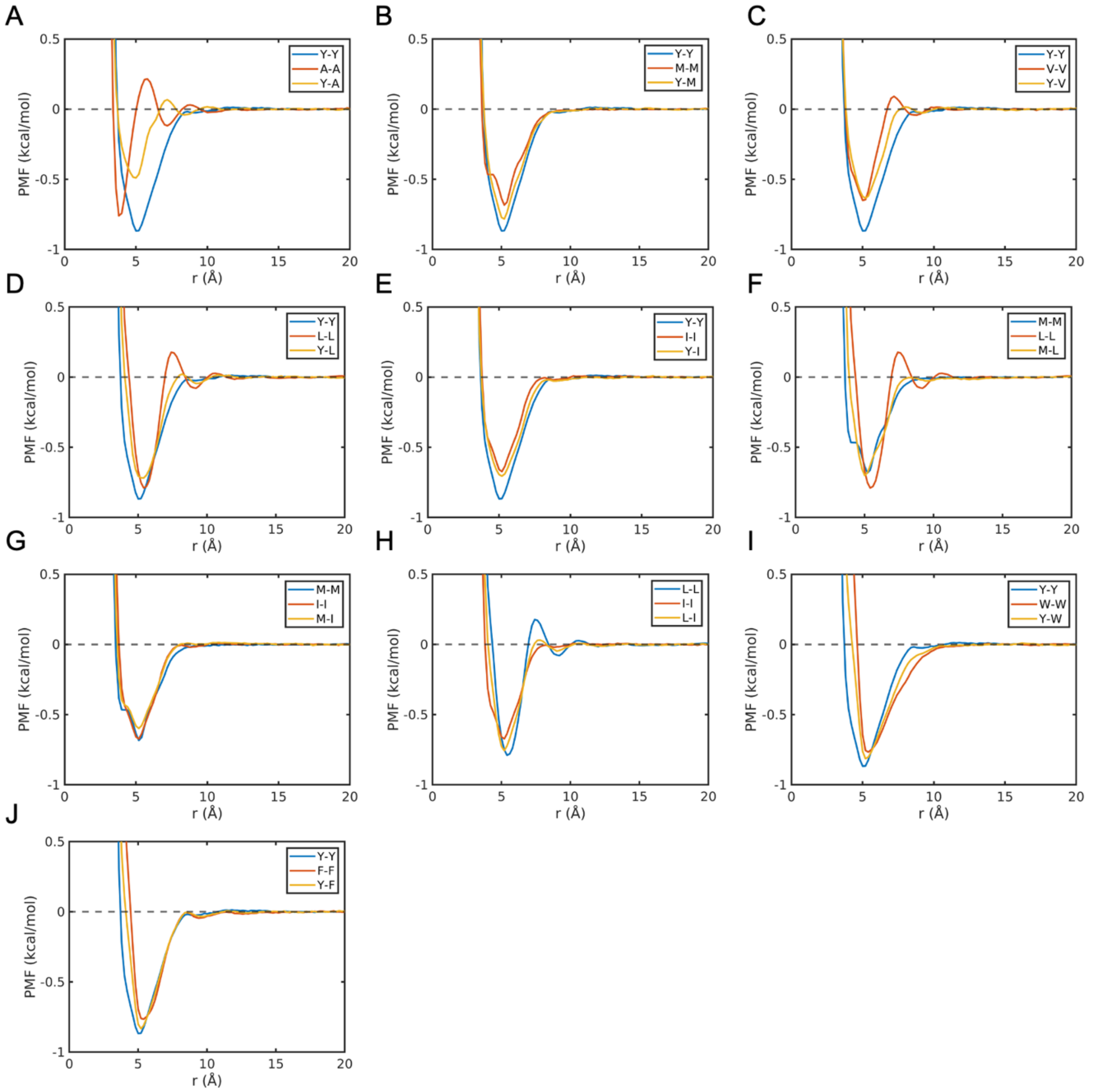

Figure S7. PMFs for the 'hydrophobic-hydrophobic' heterotypic interactions at 300 K. Heterotypic interactions are shown by yellow curves. For comparison, the corresponding homotypic interactions are shown in blue and red.

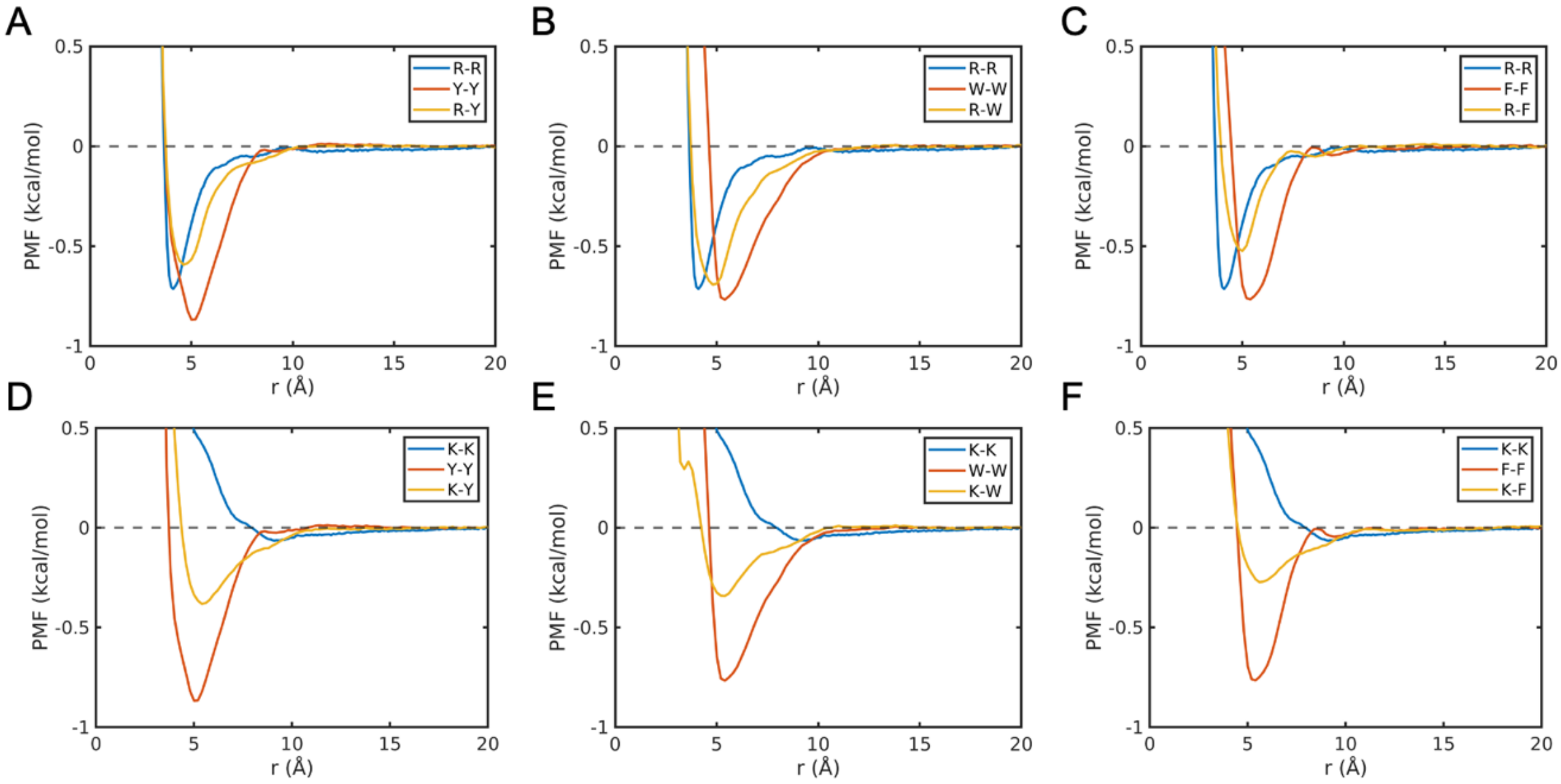

Figure S8. PMFs for the ‘cation-aromatic’ heterotypic interactions at 300 K. Heterotypic interactions are shown by yellow curves. For comparison, the corresponding homotypic interactions are shown in blue and red.

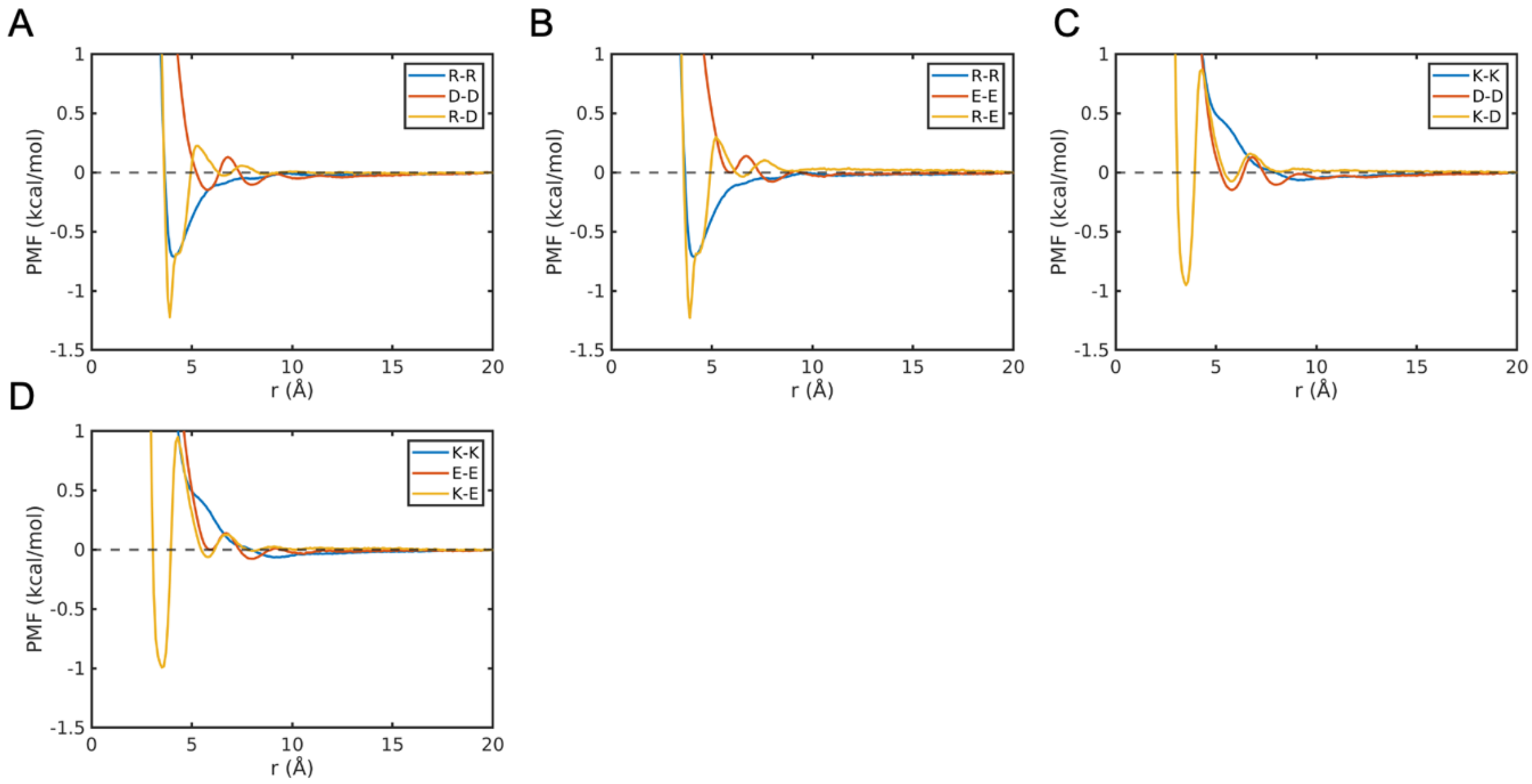

Figure S9. PMFs for the ‘charged-charged’ heterotypic interactions at 300 K. Heterotypic interactions are shown by yellow curves. For comparison, the corresponding homotypic interactions are shown in blue and red.

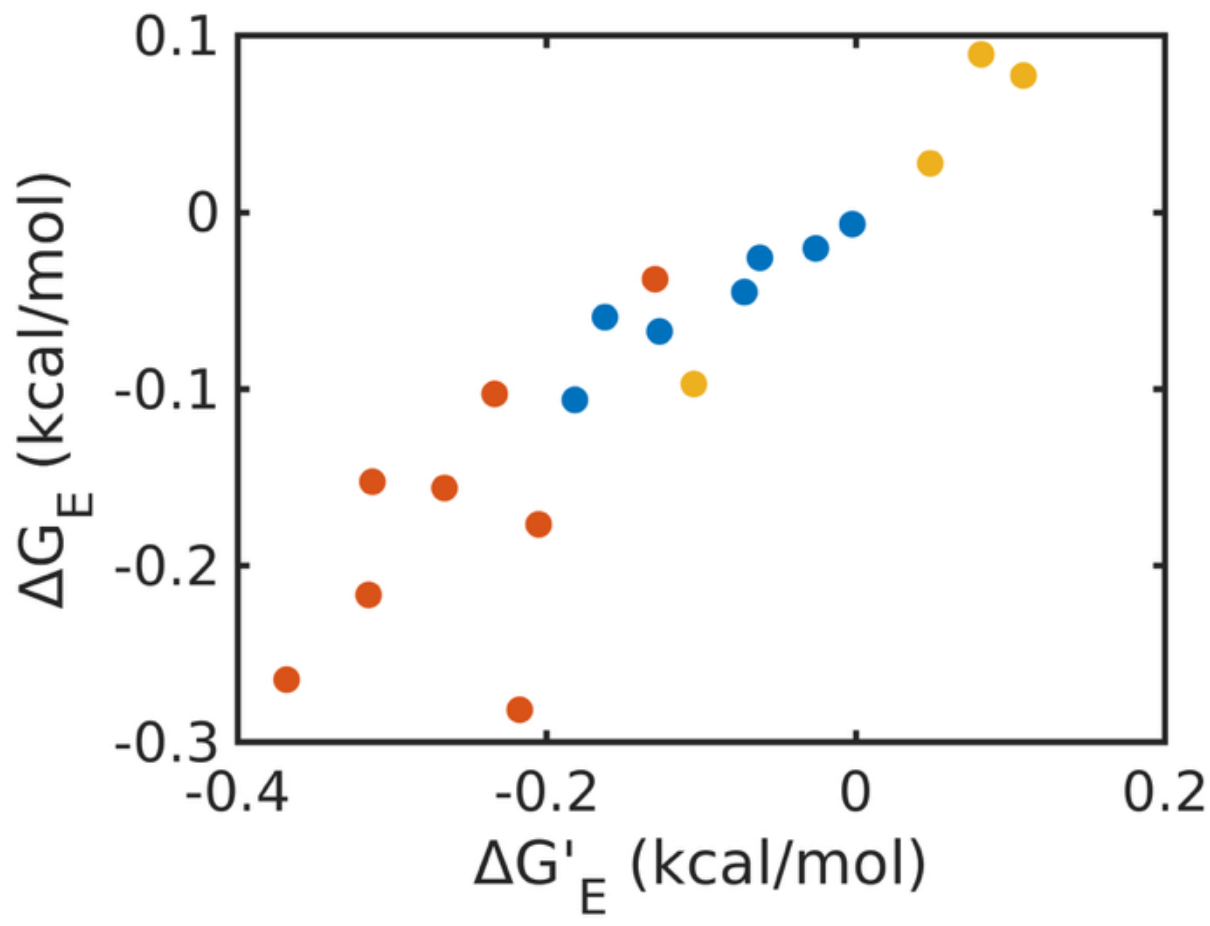

Figure S10. Excess free energies calculated using the molecular-specific cutoff, $\Delta G'_E$, at 300 K correlates strongly with those derived using the 10 Å cutoff.

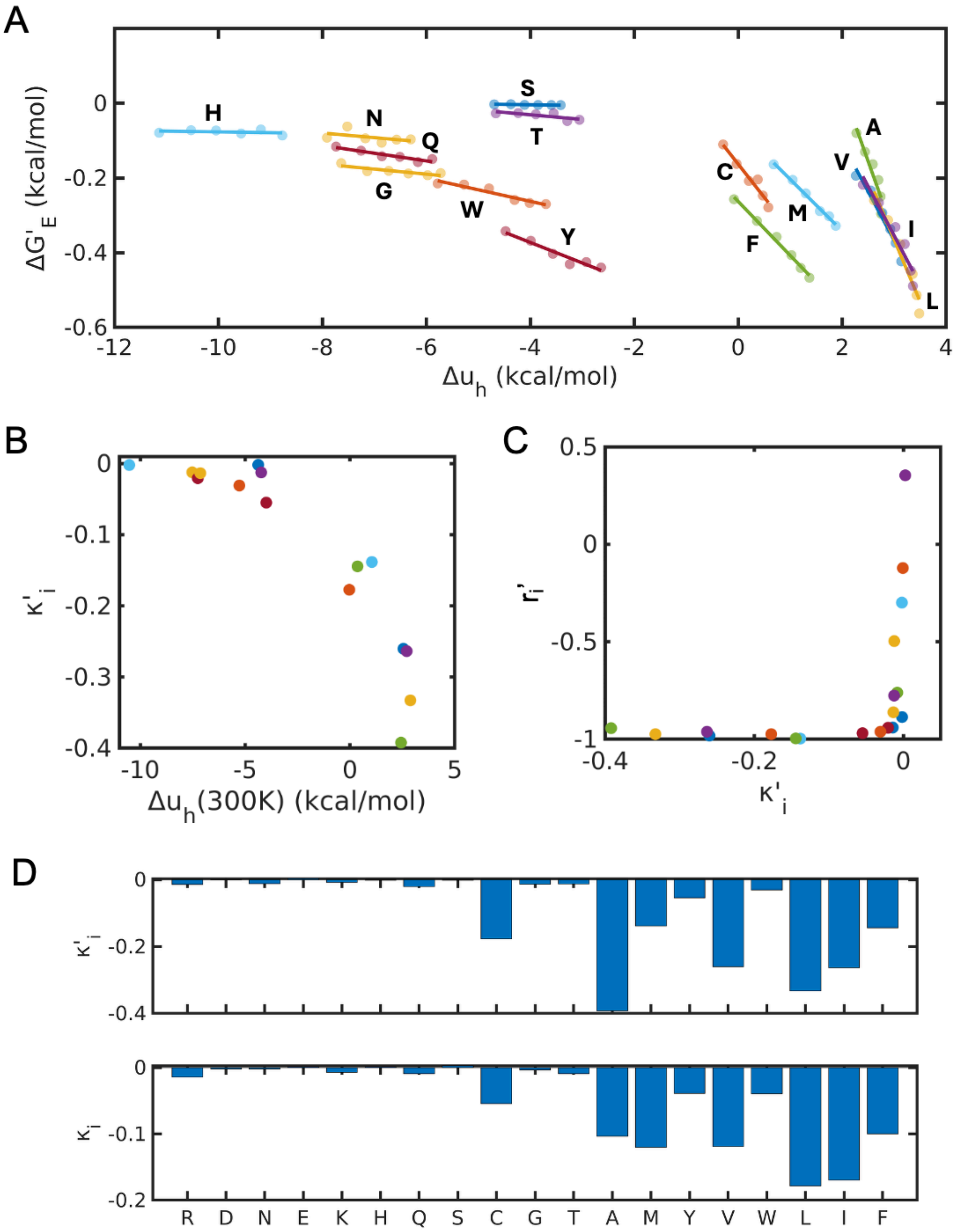

Figure S11. Excess free energies $\Delta G_E'$ exhibit linear relationship with hydration free energies Δu_h.(A) $\Delta G_E'$ versus Δu_h plot for each analogue, (B) κ_i' as a function of hydration free energies at 300 K $\Delta u_h(300K)$. (C) Dependence of the correlation coefficients r_i' on the slope κ_i. (D) Comparison between κ_i' and κ_i.

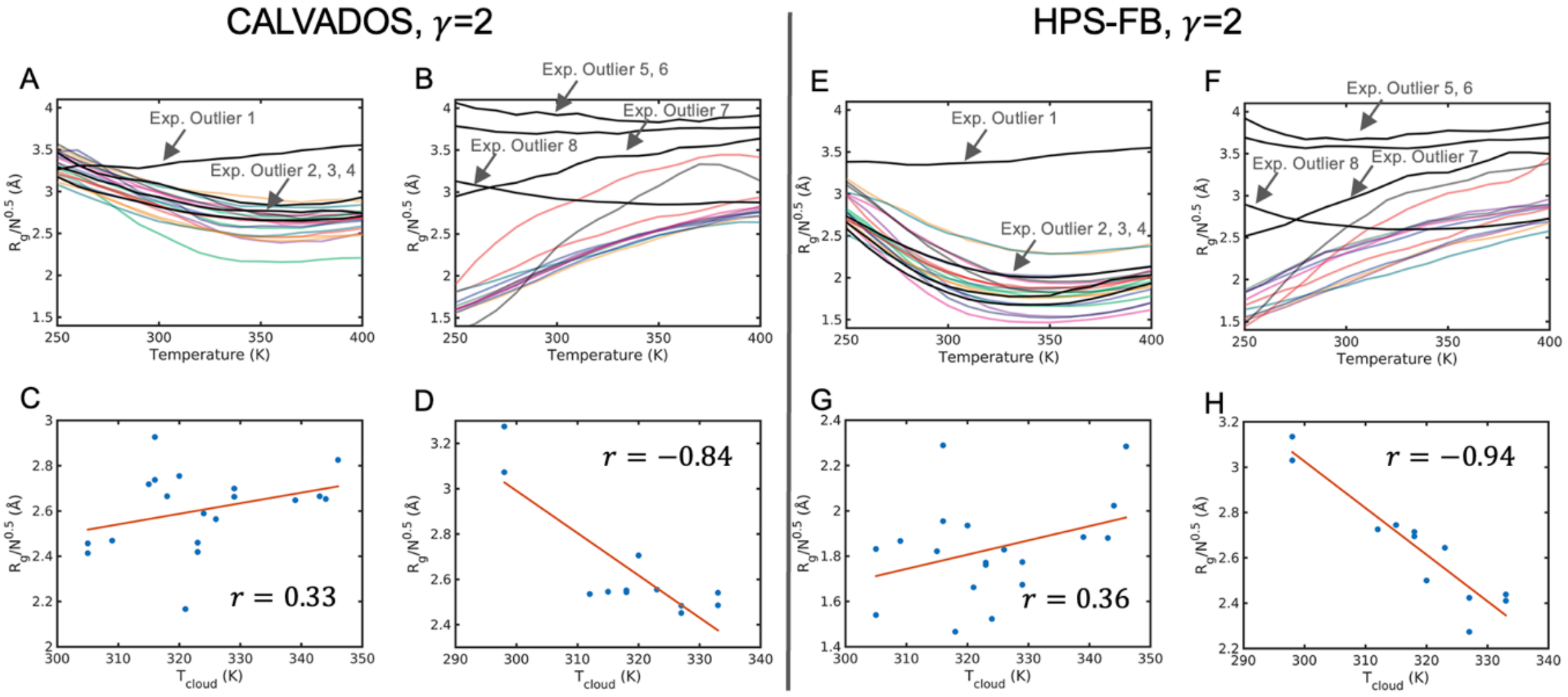

Figure S12. Performance of TEA-augmented CALVADOS and HPS-FB models ($\gamma = 2$). Comparison of simulated single-chain R_g trends for ELP (LCST-type) and RLP (UCST-type) sequences. The models successfully distinguish between the two phase behaviors and identify experimental outliers (indicated by arrows).

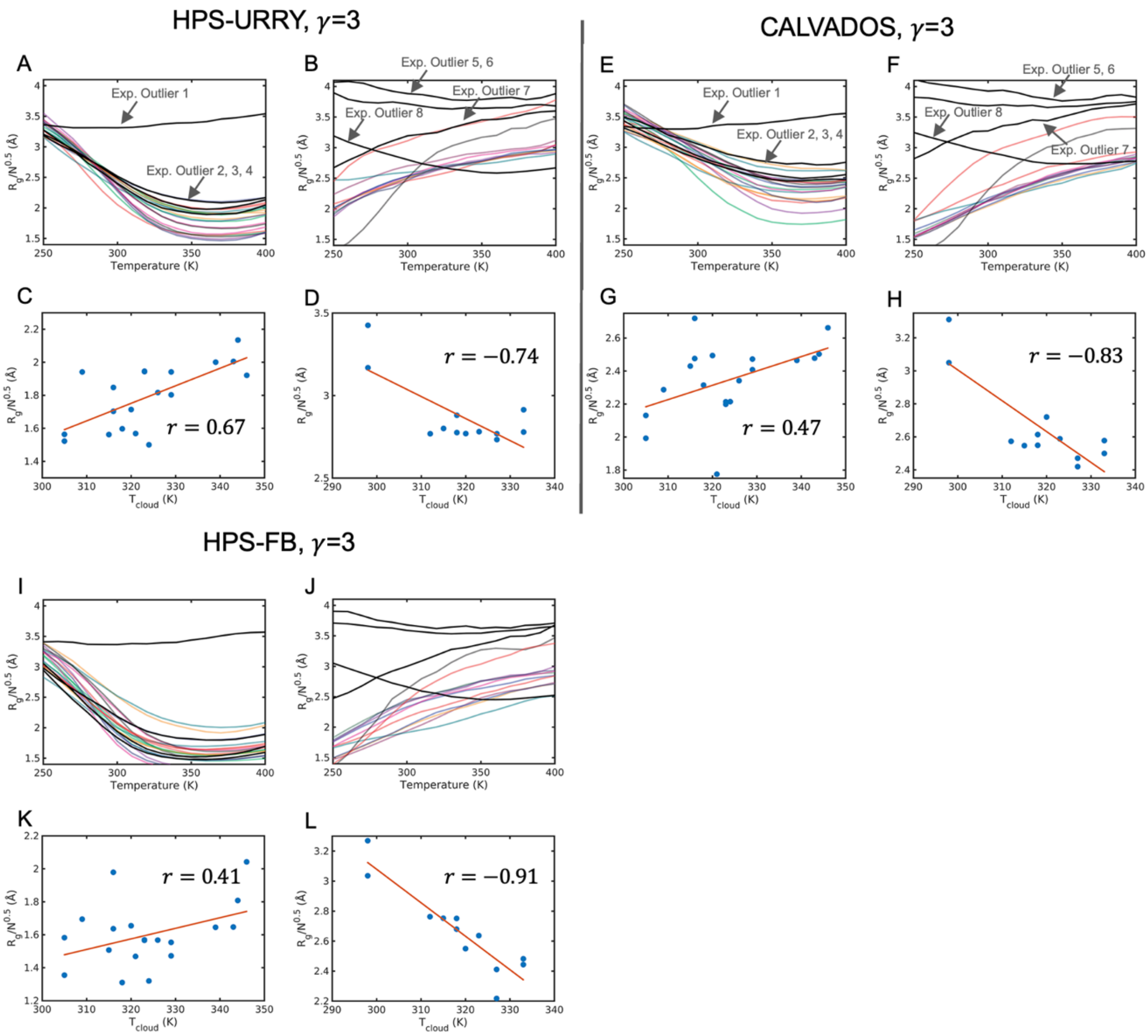

Figure S13. Performance of TEA-augmented CALVADOS and HPS-FB models ($\gamma = 3$). Comparison of simulated single-chain R_g trends for ELP (LCST-type) and RLP (UCST-type) sequences. The models successfully distinguish between the two phase behaviors and identify experimental outliers (indicated by arrows).

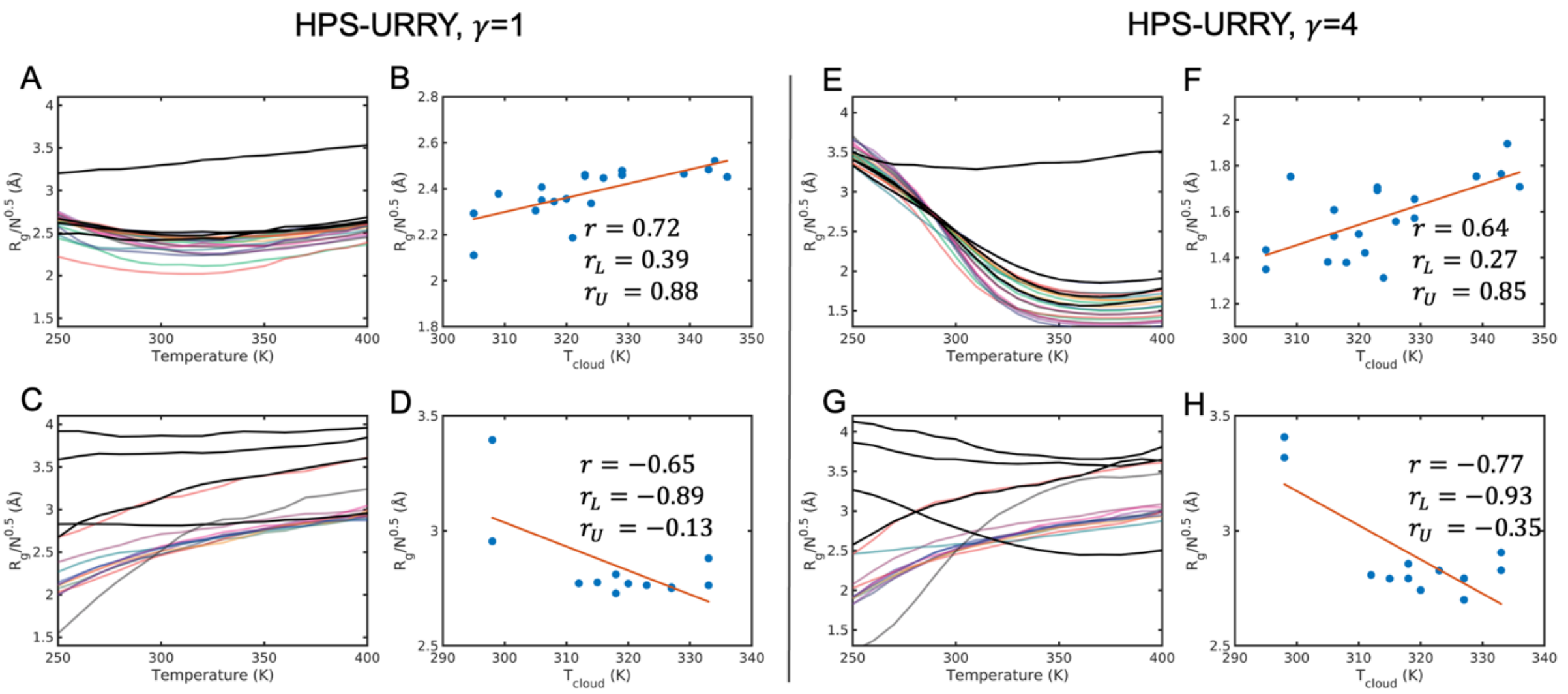

Figure S14. Performance of TEA-augmented HPS-Urry models with $\gamma = 1$ and $\gamma = 4$. Comparison of simulated single-chain Rg trends for ELP (LCST-type) and RLP (UCST-type) sequences. The models successfully distinguish between the two phase behaviors and identify experimental outliers (indicated by arrows).

HPS-URRY, γ=3

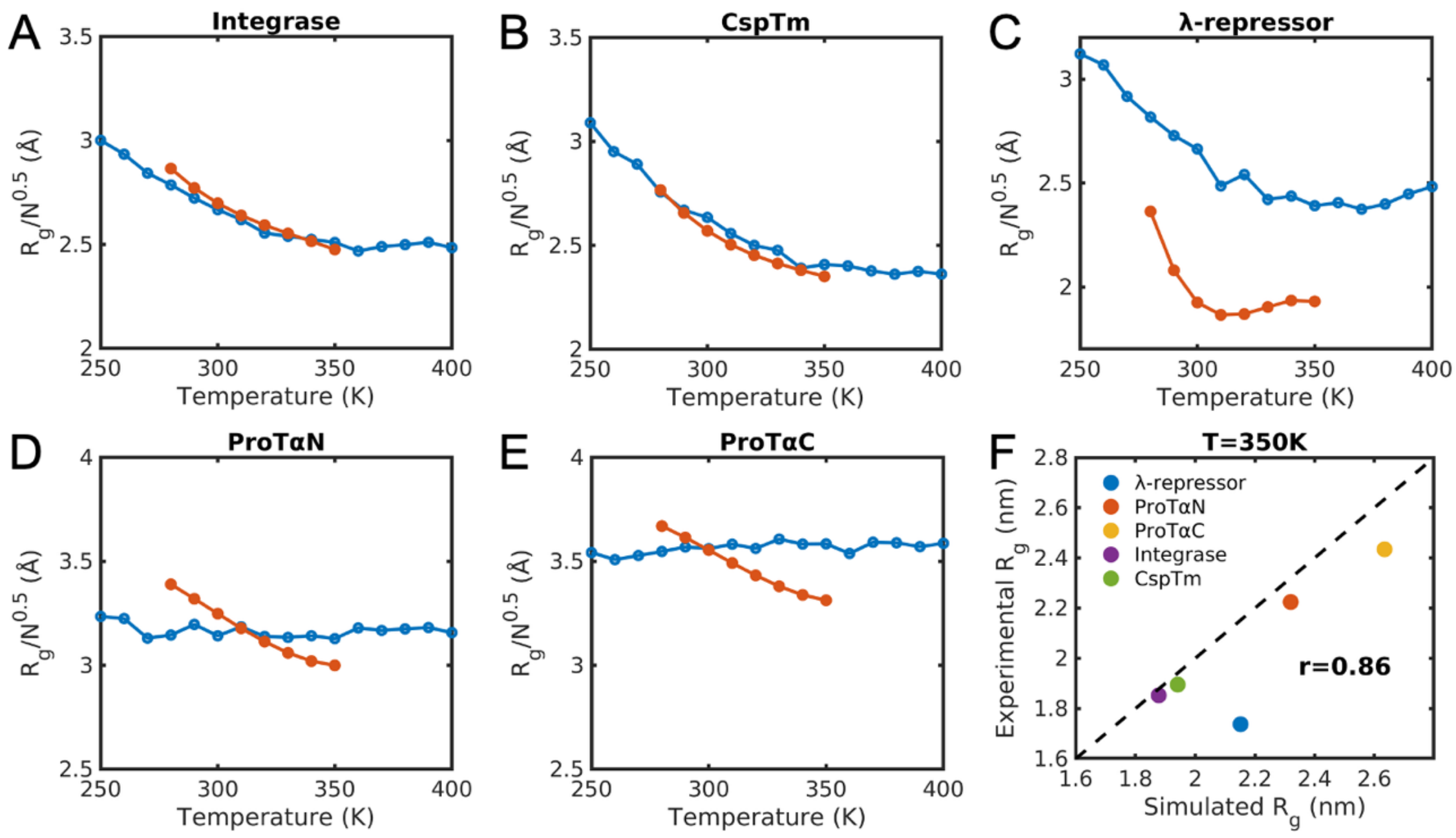

Fig. S15. Comparison of simulated and experimental single-chain dimensions for five IDPs exihibiting LCST-type phase behavior. Temperature-dpendent R_g values from TEA-augmented HPS-Urry model with $\gamma = 3$ (blue curves) are compared with experimental measurements from Wuttke et al (red curves).

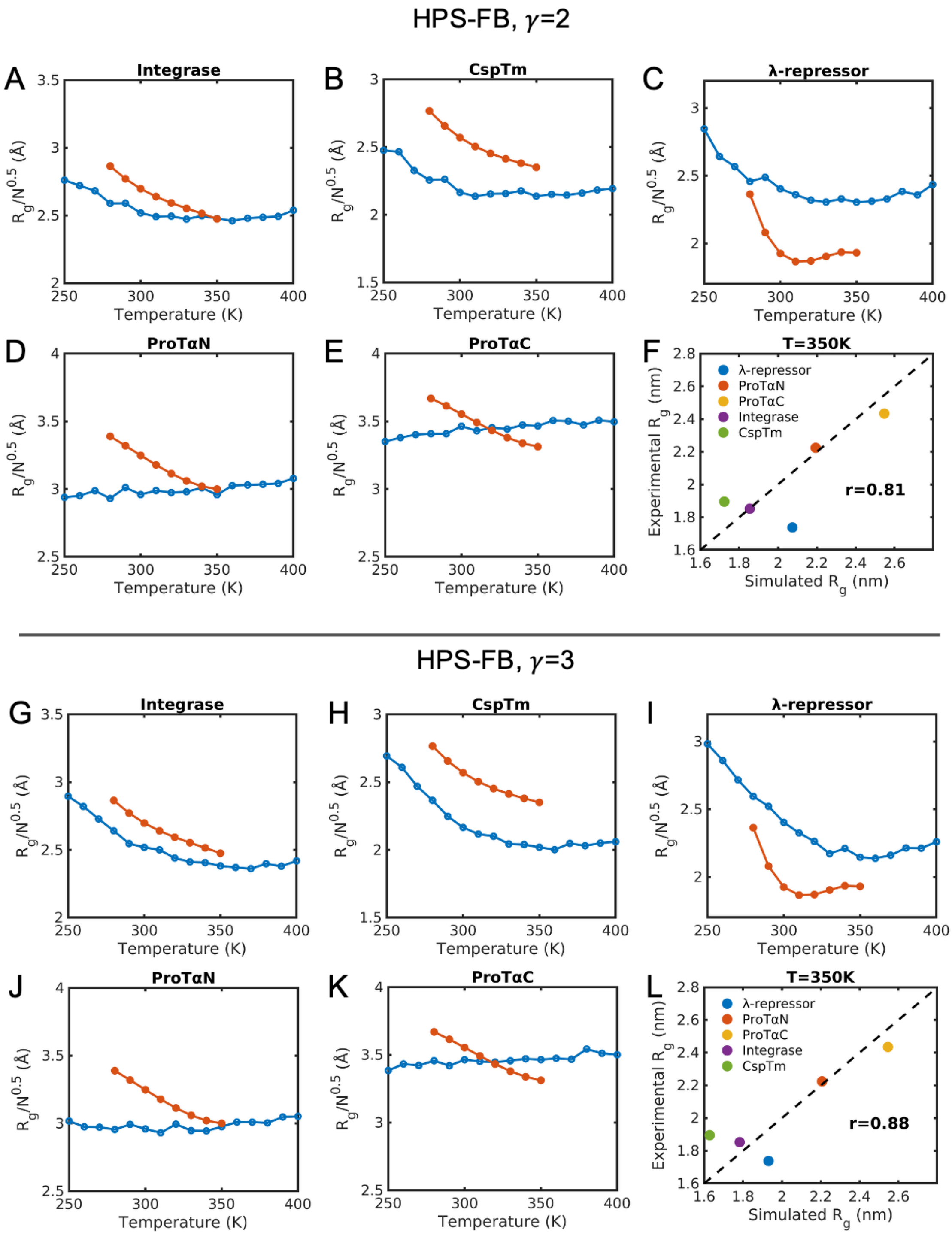

Figure S16. Comparison of simulated and experimental single-chain dimensions for five IDPs exihibiting LCST-type phase behavior. Temperature-dpendent R_g values from TEA-augmented HPS-FB model (blue curves) are compared with experimental measurements from Wuttke et al (red curves).

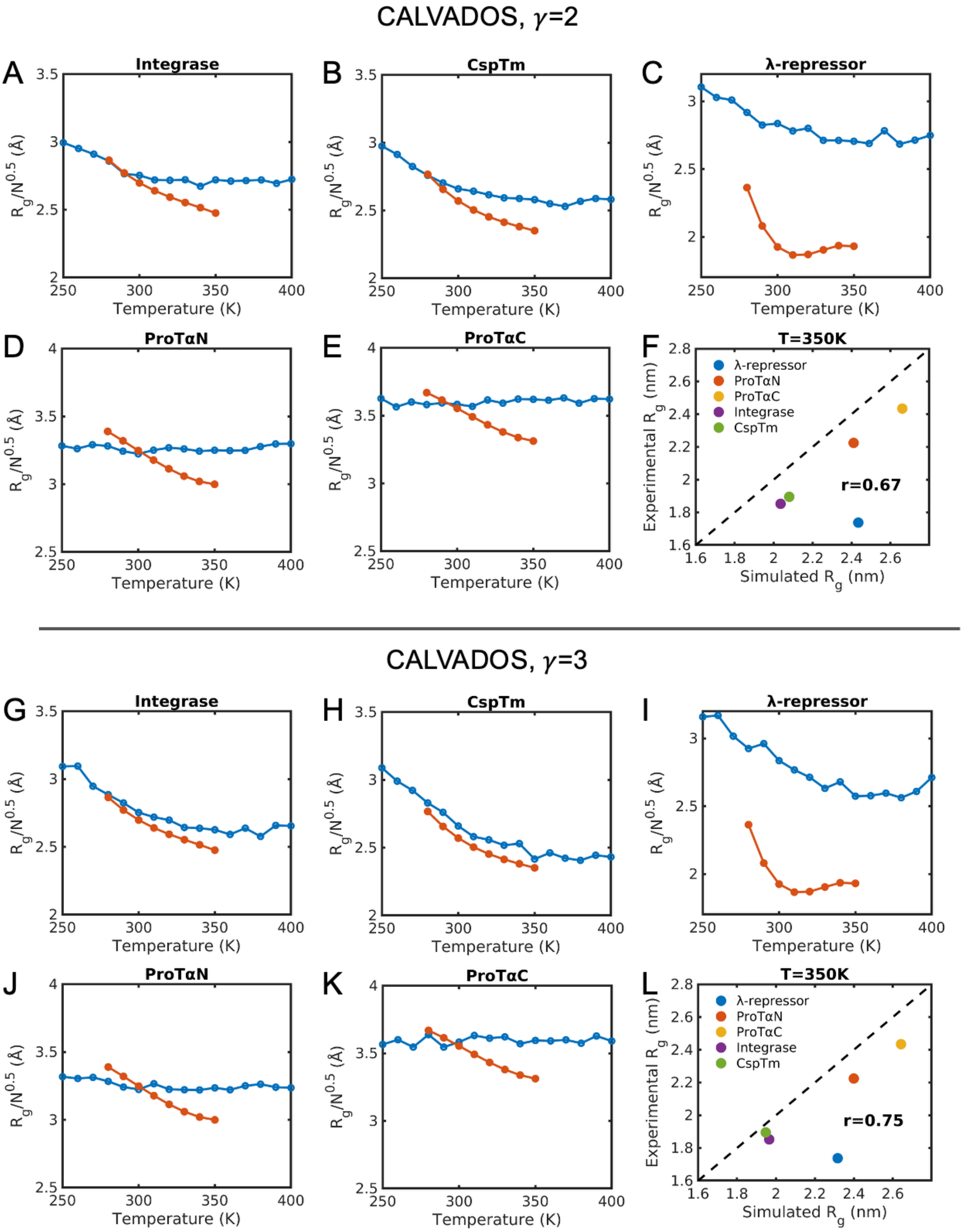

Figure S17. Comparison of simulated and experimental single-chain dimensions for five IDPs exihibiting LCST-type phase behavior. Temperature-dpendent R_g values from TEA-augmented CALVADOS model (blue curves) are compared with experimental measurements from Wuttke et al (red curves)

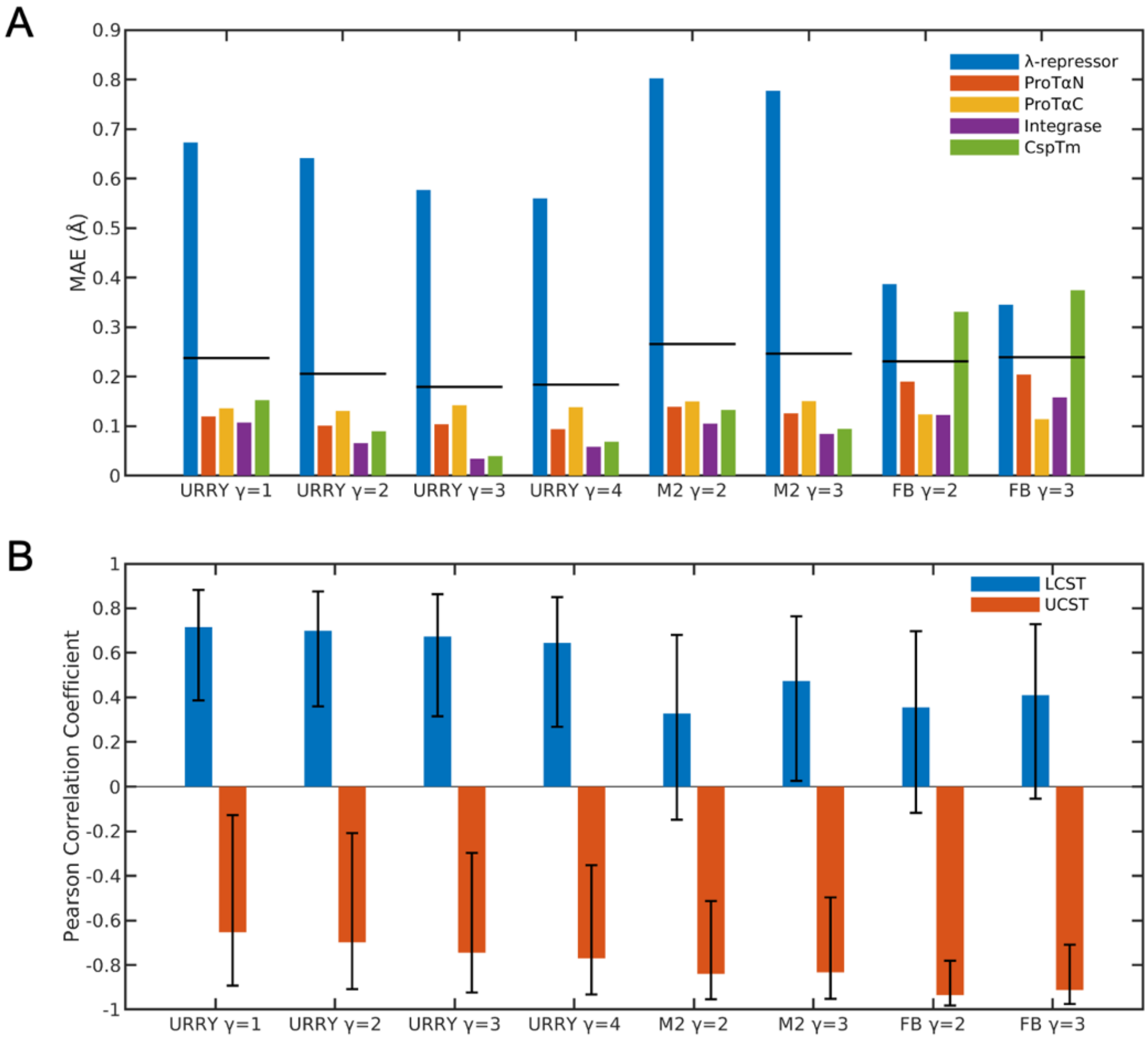

Figure S18. Comparison between simulated protein single-chain data with experimental results. (A) The mean absolute error (MAE) against single-chain dimensions from Wuttke et al. The black lines represent the average MAE values for the five proteins. (B) Pearson correlation coefficients between simulated R_g values and experimental cloud point temperatures for ELP and RLP sequences. The error bar indicates the upper and lower bounds for a 95% confidence interval, calculated using the *corrcoef* function in Matlab.

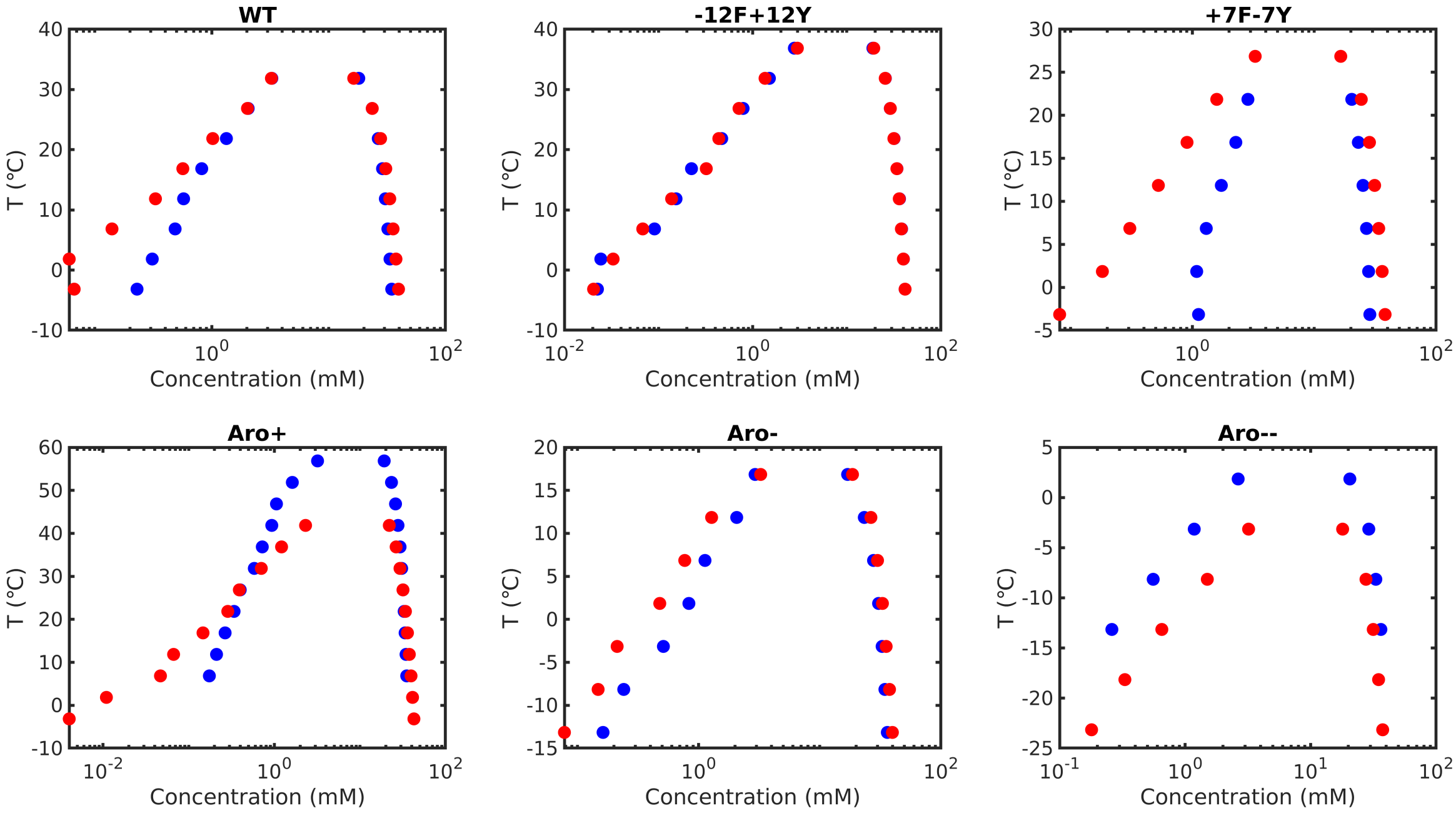

Figure S19. Impact of temperature-dependent energetics on phase diagrams. Comparison of phase diagrams from TEA-augmented HPS-Urry model (blue) versus the HPS-Urry model (red) for A1-LCD and its variants.

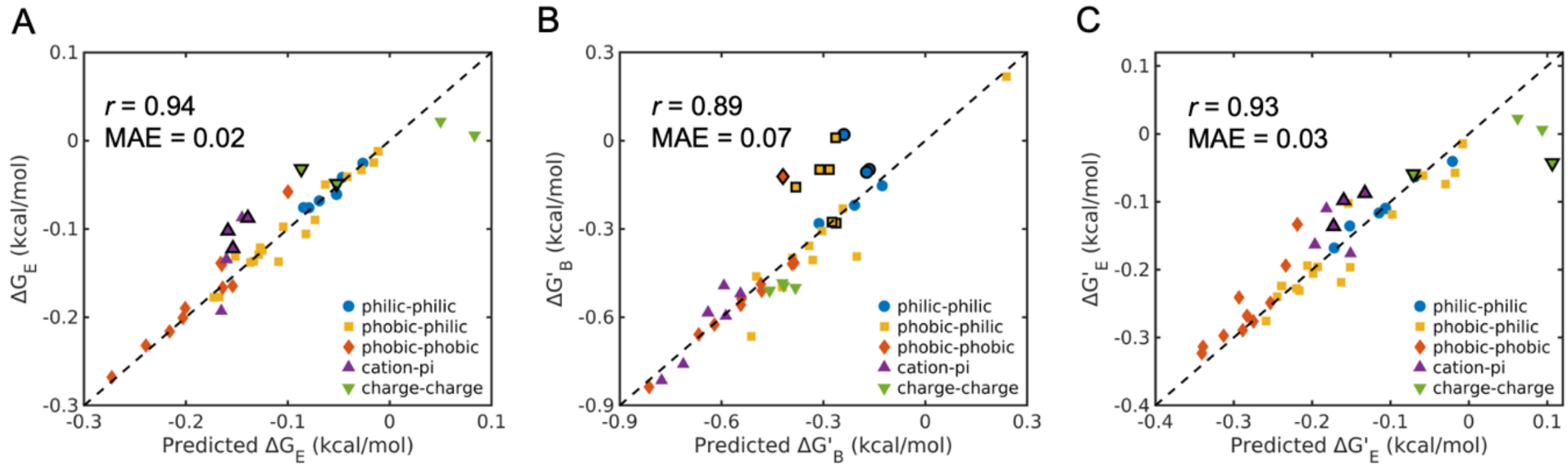

Figure S20. Predicting heterotypic interactions using the geometric mean. (A) excess free energies $\boldsymbol{\Delta G_E}$ calculated using a uniform cutoff of 10 Å, (B) excess free energies $\boldsymbol{\Delta G'_E}$ caculated using molecule-specific cutoffs, and (C) binding free energies $\boldsymbol{\Delta G'_B}$ calculated using molecule-specific cutoffs. Molecule-specific cutoff values are listed in Table S2 . Data points are colored by interaction type: hydrophilic-hydrophilic (blue), hydrophobic-hydrophilic (red), hydrophobic-hydrophobic (yellow), cation-$\boldsymbol{\pi}$ (purple), and charge-charge (green). The dashed line represents perfect agreement (y = x). The data points with black edges indicate the pairs where $\boldsymbol{\Delta G_{AA}}$ and $\boldsymbol{\Delta G_{BB}}$ possess different signs.

Hydrophobic analogues

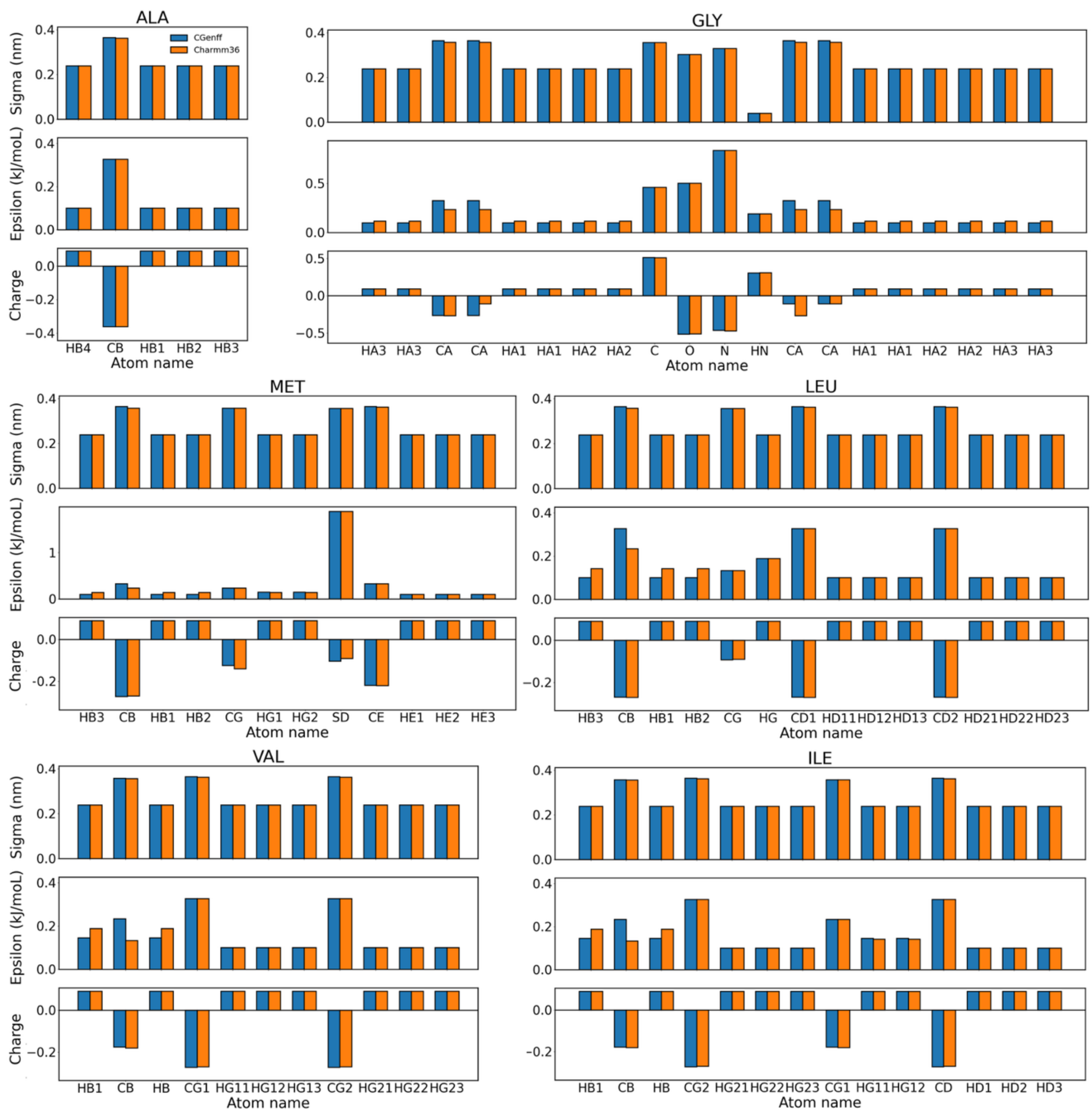

Figure S21. Comparison of the sigma, epsilon, and partial charges in CGenFF (blue) and CHARMM36m protein force fields (red) for the hydrophobic analogues.

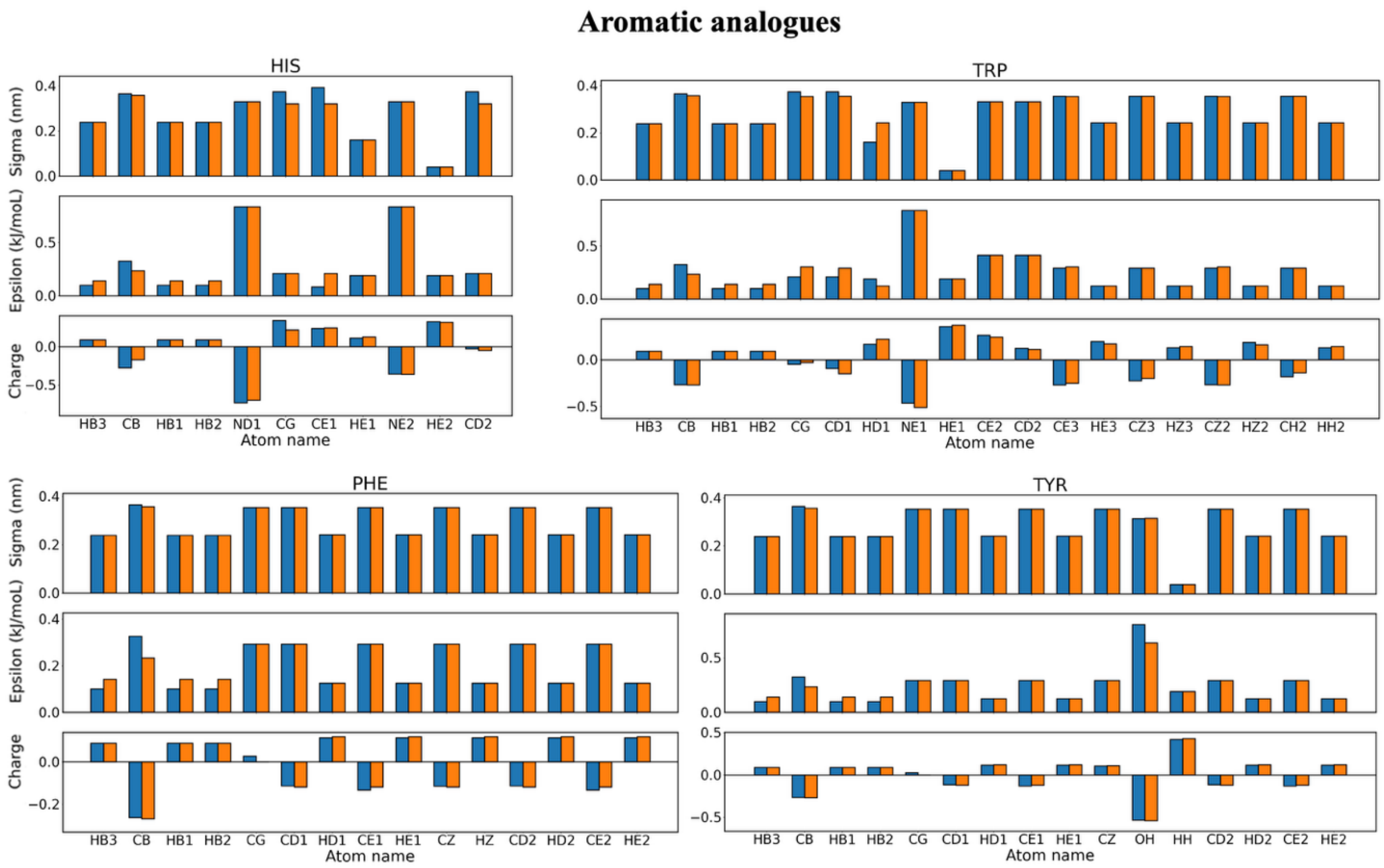

Figure S22. Comparison of the sigma, epsilon, and partial charges in CGenFF (blue) and CHARMM36m protein force fields (red) for the aromatic analogues.

Hydrophilic analogues

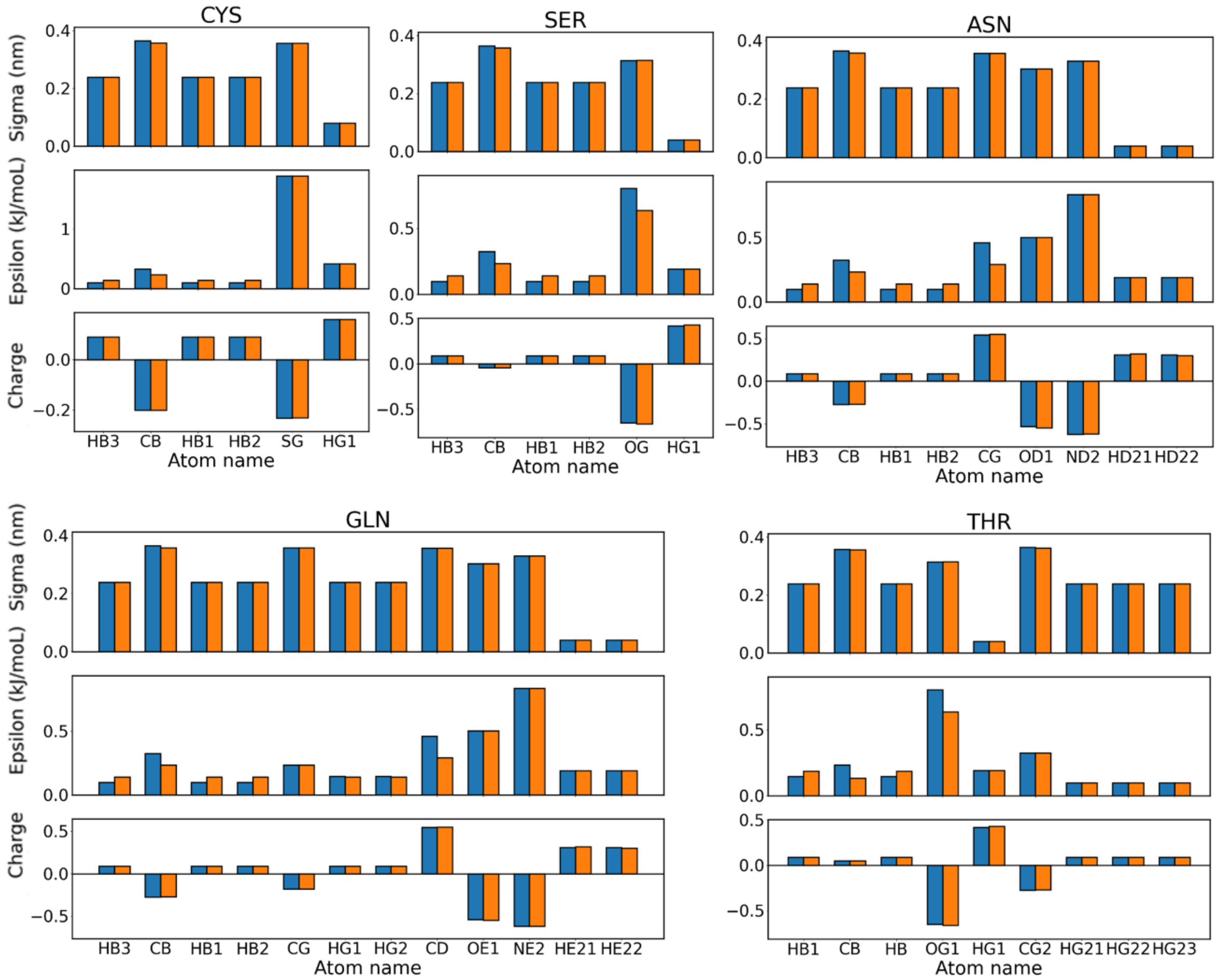

Figure S23. Comparison of the sigma, epsilon, and partial charges in CGenFF (blue) and CHARMM36m protein force fields (red) for the hydrophilic analogues.

Charged analogues

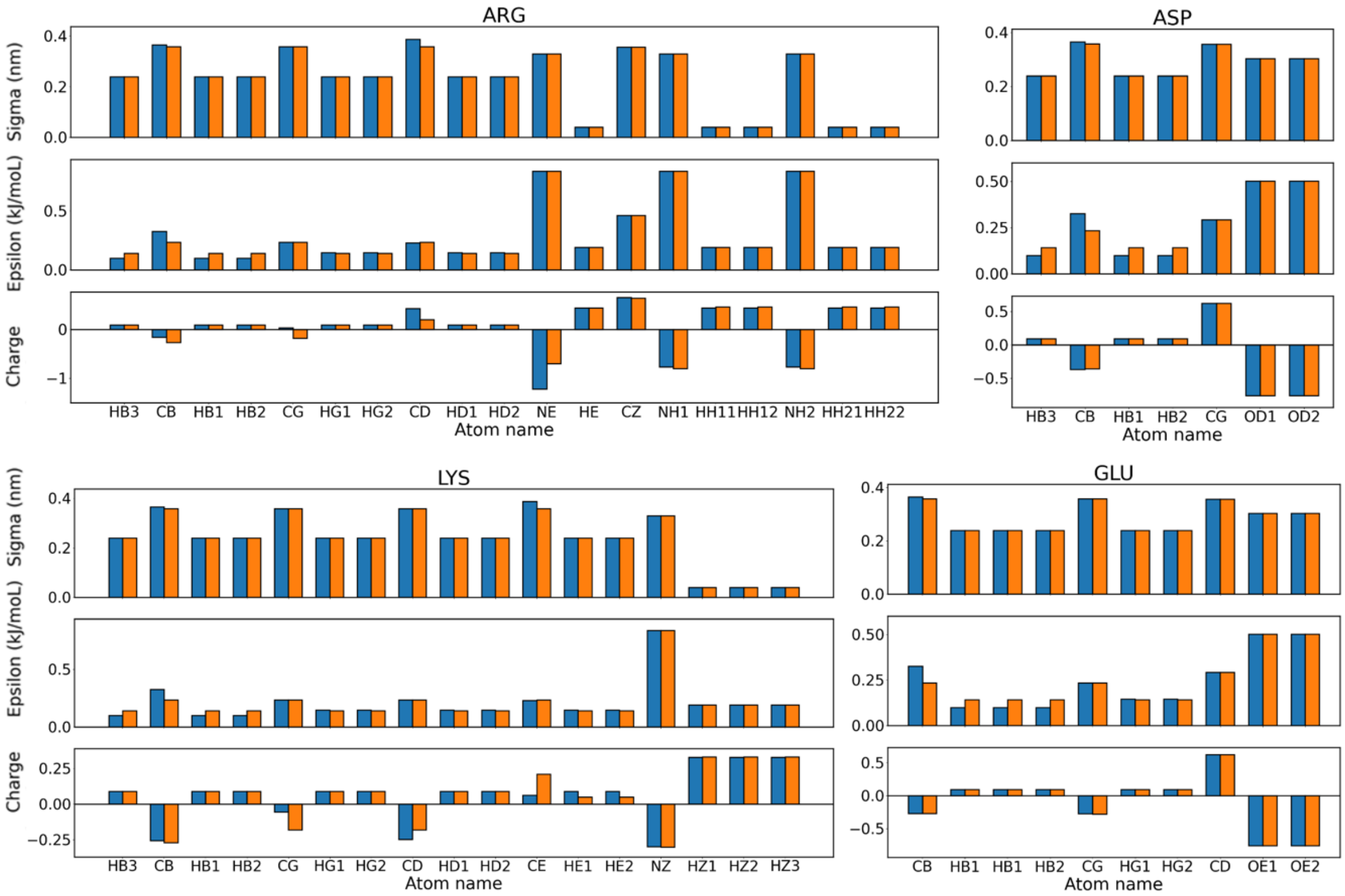

Figure S24. Comparison of the sigma, epsilon, and partial charges in CGenFF (blue) and CHARMM36m protein force fields (red) for the charged analogues.
